# Molecular dynamics and structure function analysis show that substrate binding and specificity are major forces in the functional diversification of Eqolisins

**DOI:** 10.1186/s12859-018-2348-2

**Published:** 2018-09-24

**Authors:** Nicolás Stocchi, María Victoria Revuelta, Priscila Ailín Lanza Castronuovo, D. Mariano A. Vera, Arjen ten Have

**Affiliations:** 1 Instituto de Investigaciones Biológicas (IIB-CONICET-UNMdP), Facultad de Ciencias Exactas y Naturales, Universidad Nacional de Mar del Plata, CC 1245, 7600 Mar del Plata, Argentina; 2 QUIAMM-INBIOTEC-CONICET, Department of Chemistry – Facultad de Ciencias Exactas y Naturales, Universidad Nacional de Mar del Plata, Funes 3350, 7600 Mar del Plata, Argentina; 3 Pressent address: Department of Medicine, Hematology and Oncology Division, Weill Cornell Medicine, New York, NY 10065 USA

**Keywords:** Structure-function prediction, Functional redundancy and diversification, Molecular dynamics, Acid protease, Eqolisin, Glutamic peptidase

## Abstract

**Background:**

Eqolisins are rare acid proteases found in archaea, bacteria and fungi. Certain fungi secrete acids as part of their lifestyle and interestingly these also have many eqolisin paralogs, up to nine paralogs have been recorded. This suggests a process of functional redundancy and diversification has occurred, which was the subject of the research we performed and describe here.

**Results:**

We identified eqolisin homologs by means of iterative HMMER analysis of the NR database. The identified sequences were scrutinized for which new hallmarks were identified by molecular dynamics simulations of mutants in highly conserved positions, using the structure of an eqolisin that was crystallized in the presence of a transition state inhibitor. Four conserved glycines were shown to be important for functionality. A substitution of W67F is shown to be accompanied by the L105W substitution. Molecular dynamics shows that the W67 binds to the substrate via a π-π stacking and a salt bridge, the latter being stronger in a virtual W67F/L105W double mutant of the resolved structure of Scytalido-carboxyl peptidase-B (PDB ID: 2IFW). Additional problematic mutations are discussed. Upon sequence scrutiny we obtained a set of 233 sequences that was used to reconstruct a Bayesian phylogenetic tree. We identified 14 putative specificity determining positions (SDPs) of which four are explained by mere structural explanations and nine seem to correspond to functional diversification related with substrate binding and specificity. A first sub-network of SDPs is related to substrate specificity whereas the second sub-network seems to affect the dynamics of three loops that are involved in substrate binding.

**Conclusion:**

The eqolisins form a small superfamily of acid proteases with nevertheless many paralogs in acidic fungi. Functional redundancy has resulted in diversification related to substrate specificity and substrate binding.

**Electronic supplementary material:**

The online version of this article (10.1186/s12859-018-2348-2) contains supplementary material, which is available to authorized users.

## Background

### Acid proteases

The three major families of acid or carboxyl peptidases recognized by MEROPS [1] are the well studied, pepstatin-sensitive, eukaryotic aspartic proteinases (A01, APs, for review see [2]), part of the aspartic proteinase clan A; the more recently identified sedolisins (S53) and the also novel eqolisins or glutamic peptidases (G01), both recently reviewed [3]. Both sedolisins and eqolisins were first thought to be pepstatin-insensitive AP variants but the structures that were resolved showed they are unrelated. The rather recent discovery of these enzymes means we have relatively little knowledge. Since both sedolisins and eqolisins are typically active below pH 4, they are of fundamental and industrial interest. Here we study the eqolisins by means of molecular evolution and dynamics, a study on sedolisins was recently reported elsewhere [4].

### Eqolisins are glutamic peptidases

Eqolisins have been described in archaea, bacteria [5] and fungi but are not found in any non-fungal eukaryote [6]. Furthermore, eqolisins seem to have a rather unique and simple fold, which makes them straightforward targets for structure-function prediction. Eqolisins are endopeptidases synthesized as a preproprecursor proteins. The preprosegments are approximately 55 amino acids in length and the prosegments are rich in positively charged residues. Scytalido-carboxyl peptidase-B (SCP-B) from *Scytalidium lignicolum* is the enzyme that first described the eqolisin peptidase family and its structure has been resolved (PDB ID: 2IFW) [7]. Point mutation analyses revealed its catalytic site is formed by a catalytic dyad (Q53, E136), hence the name EQolisin. SCP-B has a narrow substrate specificity, with preference for small, basic residues, rather than the typical hydrophobic residues preferred by APs [2]. D43 is a residue that is important for SCP-B structure since the D43A mutant has approximately 20% of the original activity [8, 9]. Other characterized eqolisins are *Aspergillus niger* carboxyl peptidase (ANCP) [10], which 3D structure has also been resolved (1Y43) [11], *Sclerotinia sclerotiorum* carboxyl peptidase [12], *Cryphonectria parasitica* peptidases B and C [13], *Talaromyces emersonii* carboxyl peptidase TGP1 [14] and bacterial *Alicyclobacillus* sp. pepG1 [5].

**Fig. 1:**
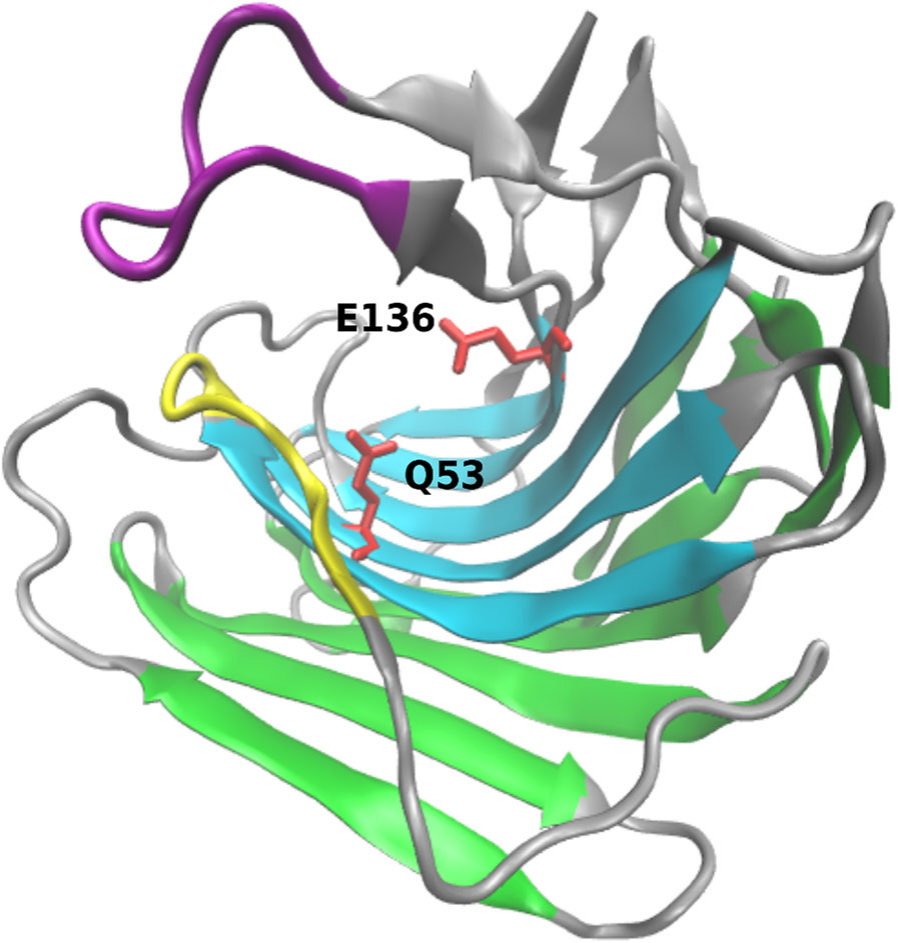
Structure of SCP-B Eqolisin. Eqolisins consist of two pleated β-sheets (Cyan and green for inner and outer sheet, respectively) that fold into a double convex halfpipe. E136 and Q53 (red licorice) stick into the binding cleft and form the catalytic site. The β-loop (purple) and 70’s-loop (yellow) have been shown to migrate inwards during substrate binding

Eqolisins are composed of two seven-stranded anti-parallel β-sheets that fold in parallel and bend to form a structure resembling a half-pipe that forms the binding cleft (See Fig. 1). The catalytic Q53 and E136 (numbering according to SCP-B structure 2IFW) stick into the binding cleft. Pillai and coworkers [15] described the 70’s loop (Tyr71-Gly80) and the β-loop (Cys141-Cys148) that, upon interaction with a transition state inhibitor, appear to move inwards and probably play a role in substrate binding. They also indicated eqolisins show structural similarity with the carbohydrate-binding concanavalin A-like lectins/glucanases superfamily. Furthermore, residues Y64 to Y71 were shown to be highly conserved across all members of the G1 family [15].

### Fungal Eqolisins have undergone a process of functional redundancy and diversification

Since eqolisins have only been recently described their functional characterization is incomplete. *T. emersonii*’s eqolisin gene *tgp1* was shown to be induced in the presence of an extracellular protein source, displays a broad specificity, is the most abundant protease in its secretome and essential for fungal growth [14]. Poussereau and collaborators described the importance of an eqolisin from plant pathogen *S. sclerotiorum* in the sunflower cotyledon infection process, suggesting a role in pathogenesis [12]. Closely related plant pathogenic fungus *Botrytis cinerea* contains nine paralogues, which suggests a process of functional redundancy and diversification has occurred. This is supported by the fact that *B. cinerea* and *S. sclerotiorum,* as well as for instance many *Aspergilli,* all secrete acids and have various eqolisin paralogs. Interestingly, these acid secreting fungi also show relatively many acid sedolisins but few basic subtilisins. Recently we studied the sedolisins [4], here we explore the eqolisin protein family in order to identify which residues are likely required for function and which residues could be involved in functional diversification.

## Methods

### Identification of Eqolisin homologues

A HMMER profile was made by means of *hmmbuild* using default settings and the MEROPS [1] G1 alignment of holozymes (HMMER Version 3.0 [16]). This was used to seed an iterative HMMER screen of the 107 complete proteome dataset previously used to identify Aspartic Proteases [17]. All sequences identified with an E-value smaller than the HMMER exclusion threshold of 0.01 were considered as Eqolisin homologues. Upon data acquisition, sequences were scrutinized for the presence of catalytic residues Q53 and E136; and secondary structure elements according to available resolved structures 2IFW and 1Y43. The novel dataset was then aligned by MAFFT [18] and used for iteration of *hmmbuild* and *hmmsearch* at the HMMER [19] website using the NR database. Iterations were performed until data convergence. Finally, sequences with long (> 15 aa) inserts that appeared to lack homologous counterparts in any of the collected sequences or in a homologous sequence identified by BLAST in the non redundant database of NCBI, were removed.

### MSA and phylogenetic analysis

Multiple protein sequence alignments (MSAs) were performed using MAFFT [18] with slow iteration mode. Trimming for phylogeny was performed with Block Mapping and Gathering with Entropy v1.0 (BMGE) [20] using the command options *-t AA -m BLOSUM30 -b 1 -h 0.9*. This setting did not remove subsequences corresponding to major secondary structure elements. A maximum likelihood (ML) phylogeny was built using PhyML-a-bayes [21] with the WAG model, estimated proportion of invariable sites, four rate categories, estimated gamma distribution parameter and optimized starting BIONJ tree, as determined by a prior estimation using ProtTest [22], with 10,000 bootstraps branch support measure. The ML tree was used as starting tree for Bayesian analysis using MrBayes [23]. Chains were initiated with ten perturbed trees using default settings until convergence was reached. Convergence was tested when split frequency was below 0.01 using Awty [24]. The resulting phylogenetic trees were viewed and edited with iTol version 2.0 [25] or Dendroscope [26].

### Molecular modeling and dynamics

#### Mutant models for static analysis were made by I-Tasser [27]

The right protonation state of the wild type (WT) structural model (PDB ID: 2IFW) was determined by evaluating which possible protonation states, determined by pKa of sidechains and a pH of 4 and involvement in salt- and H-bridges, result in stable conformations. Finally 40 ns simulations of 19 different protonation states were performed (See Additional File 1). The mutants W67F, W67F-L105W, L105W, P72K and G8A-G41A-G44A-G55A (hereafter GAx4), as well as the four individual GA mutants, were prepared by replacing the side chain in the WT followed by local minimization using the AMBERTools [28] LEAP facility. As an additional check or positive control, the D43A mutant was also prepared, since the importance of D43 for functionality has already been shown [8].

The general setup for the molecular dynamics (MD) simulations was as follows: I) 2500 steps steepest descent minimization of the whole system, keeping the protein positionally restrained and embedded into a box of TIP3P water molecules with a minimum distance of 10 Å to each wall, and Cl- or Na + counter-ions to neutralize as required. II) 2500–5000 conjugate gradient minimization of the whole system. III) 150 ps slowly heating in the NTV ensemble. IV) 40 ns of simulation in the NTP ensemble, at 1 atm and 298.15 K. The procedure III-IV was repeated in three independent trajectories using the Langevin and twice the Andersen termostat/barostats [29, 30]. Then, 40 ns of NTP simulation of the WT under Andersen thermostat/barostat were used as reference for cross-correlations analysis, lowest normal modes visualization, and salt- and H-bridges inspection and comparison with the results obtained for the nine mutants discussed below. As an extra check, the WT and the mutant that showed the largest structural changes with respect to its initial structure (GAx4), were both simulated over 60 additional ns completing a set of trajectories of 100 ns; besides, the comparison with the other mutants was done using the first 40 ns. In the equilibrated system, the density slightly fluctuated around 1.019 g/mL. Electrostatic interactions were computed using the Particle Mesh Ewald (PME) method with a cutoff of 10 Å [31, 32]. Bonds involving hydrogen atoms were constrained using the SHAKE algorithm [32], allowing for an integration time step of 0.0015 ps. The integration was done using the pmemd.CUDA module of the AMBER14 program, with the ff14SB force field [33, 34]. For the inhibitor, since most residues were of proteic nature, the same force field was applied, by manually modifying the connectivity of its backbone, the residues were labeled with three capital letter in order to distinguishe them from the protein, starting from 207 to 213: ACE207, PHE208, LYS209, PHE210, PSA211, LEU212 and AAR213. The trajectories were analyzed using standard AMBER analysis tools. The criteria for analyzing the persistence of H-bonds were set to a maximum length of 3.2 Å (between the heavy atoms) and a maximum angle of 120° (donor-H-acceptor). Analysis of the hydrogen bonds, contacts persistence, mobility factors and cross correlation functions were done using ccptraj (AMBERTools 15 utilities) and VMD1.9.7 [35], which was also used for graphics rendering. Essential normal modes were calculated using the last 35 ns and processing with principal components analysis as implemented in ProDy [36].

The free energy calculations were done using the MM-PBSA module of AMBER 14 and reported for the two models applied, *i. e.* Poisson-Boltzman (PB) and Generalized Born (GB) [37, 38]. The energetic analyses were done from 5.0 to 40.0 ns of simulation as average over at least two independent trajectories.

### Additional biocomputational analyses

Sequence LOGOS were created with WebLogo [39], and structures were aligned in VMD [35] using the STAMP [40] extension. MSA quality was determined using TCS [41]. Effect of virtual mutations on enzyme function was predicted using SNAP [42].

## Results and discussion

### Sensitive identification of Eqolisin homologues likely lacks specificity

**Fig. 2:**
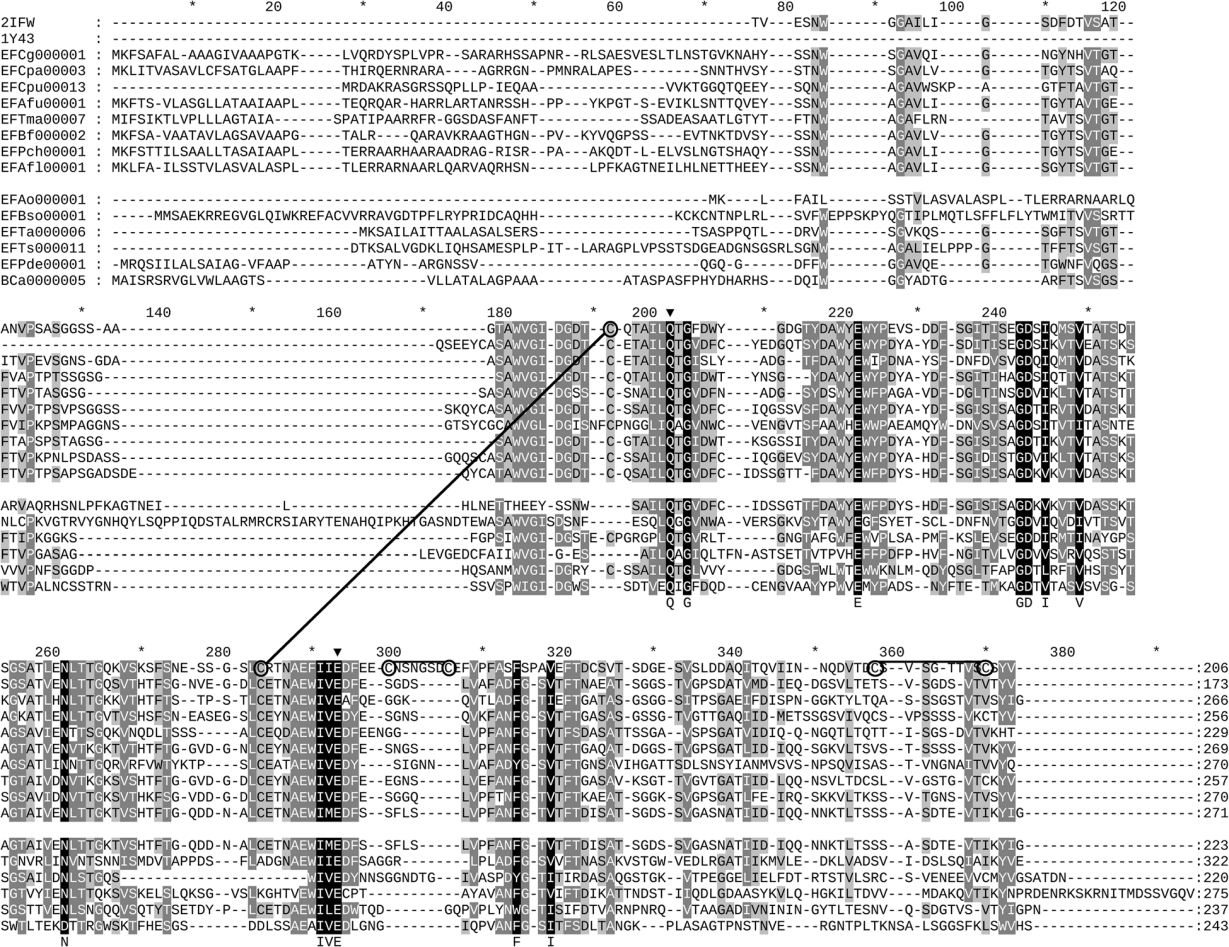
Excerpt of Multiple Sequence Alignment Eqolisins. Indicated are the sequences for which a structure has been resolved (PDB IDs 2IFW, 1Y43) as well a number of sequences selected to show overall sequence variation. The lower block contains sequences that were removed upon sequence scrutiny as described in the main text. The rulers indicate positions of hallmark residues and or positions described under the sequence scrutiny. Encircled cysteines are involved in disulfide bridges, indicated by lines. Shading indicates conservation (100% black; 80% dark-gray, 60% light-gray)

The identification of eqolisin encoding sequences was performed with HMMER [16] in order to obtain high sensitivity. A profile made from the holotype sequences of MEROPS [1] was used as a seed. Initially, a collection previously used for a study of aspartic proteases [17], of mostly fungal, but also other taxonomically well distributed eukaryotic complete proteomes were used. This resulted in few sequences, hence, the NR database was scanned and all identified sequences were combined and searched iteratively until data convergence. A complete preliminary MSA with all identified sequences can be found in Additional file 2. Then we performed a sequence hallmark scrutiny with the objective of removing problematic sequences. The sensitive HMMER search resulted in a collection of homologous sequences that may include sequences from non-functional homologs (NFHs) or incorrectly predicted gene models. These will negatively affect the construction of the MSA and therewith the eventual identification of specificity determining positions (SDPs) involved in functional diversification. Since eqolisins have been described only recently and appear restricted to fungi, bacteria and a few archaea, there is little knowledge on which residues other than the catalytic Q53 and E136 are required. Given the broad taxonomic distribution we reasoned that residues that appear highly conserved in a preliminary MSA, defined as > 95% using Genedoc’s default scheme of conserved groups, must be important and might even be required. Fig. 2 shows an excerpt of a preliminary MSA that, besides 13 strictly conserved positions, had 10 highly conserved (> 95%) positions that were all studied as part of a rigid sequence scrutiny. We performed structural analysis, including MD simulations, of apparent rare substitutions since this can corroborate if the highly conserved residues are probably strictly conserved. Additional file 3 shows a summary of additional TCS and SNAP analyses that were performed in order to provide additional evidence for problematic sequences. Note that we do not claim removed sequences correspond with NFHs.

### Wild type dynamics

We first determined the optimal protonation state. The most likely protomer at pH = 4 was chosen in terms of structure conservation of the X-ray structure (PDB-ID: 2IFW) after 40 ns of simulation. Additional file 4 A shows the comparison of the six best protomers -out of 19 possible protomers - of the WT structural model. The best state shows the lowest RMSD which remains clearly below the RMSD of other protonation states during the 40 ns of the simulations. The complete 100 ns simulation performed for the best protonation state showed an average of 0.892 Å over the whole extended simulation. In addition, the co-crystallized inhibitor perfectly conserved its structure, main contacts and conformation during the whole simulation, with an RMSD average below 0.395 Å (Additional file 4 B.). Hence, this protonation state was selected for all further analyses in which the X-ray structure of the WT was compared to the 3D models of a number of selected virtual mutants.

### Eqolisins have four highly conserved glycines that appear strictly required

Three highly conserved glycine residues, G8, G41 and G44 are found in the inner sheet together with strictly conserved G55. Since glycine is small and flexible we envisaged these glycines might play a crucial role in enzyme dynamics. EFAo000001 has G41E and G44S substitutions with surrounding subsequences that substantially differ from the otherwise highly conserved part of the MSA (Fig. 2). Hence, this sequence unlikely encodes for a functional eqolisin and G41 as well as G44 might be considered as strictly conserved.

**Fig. 3:**
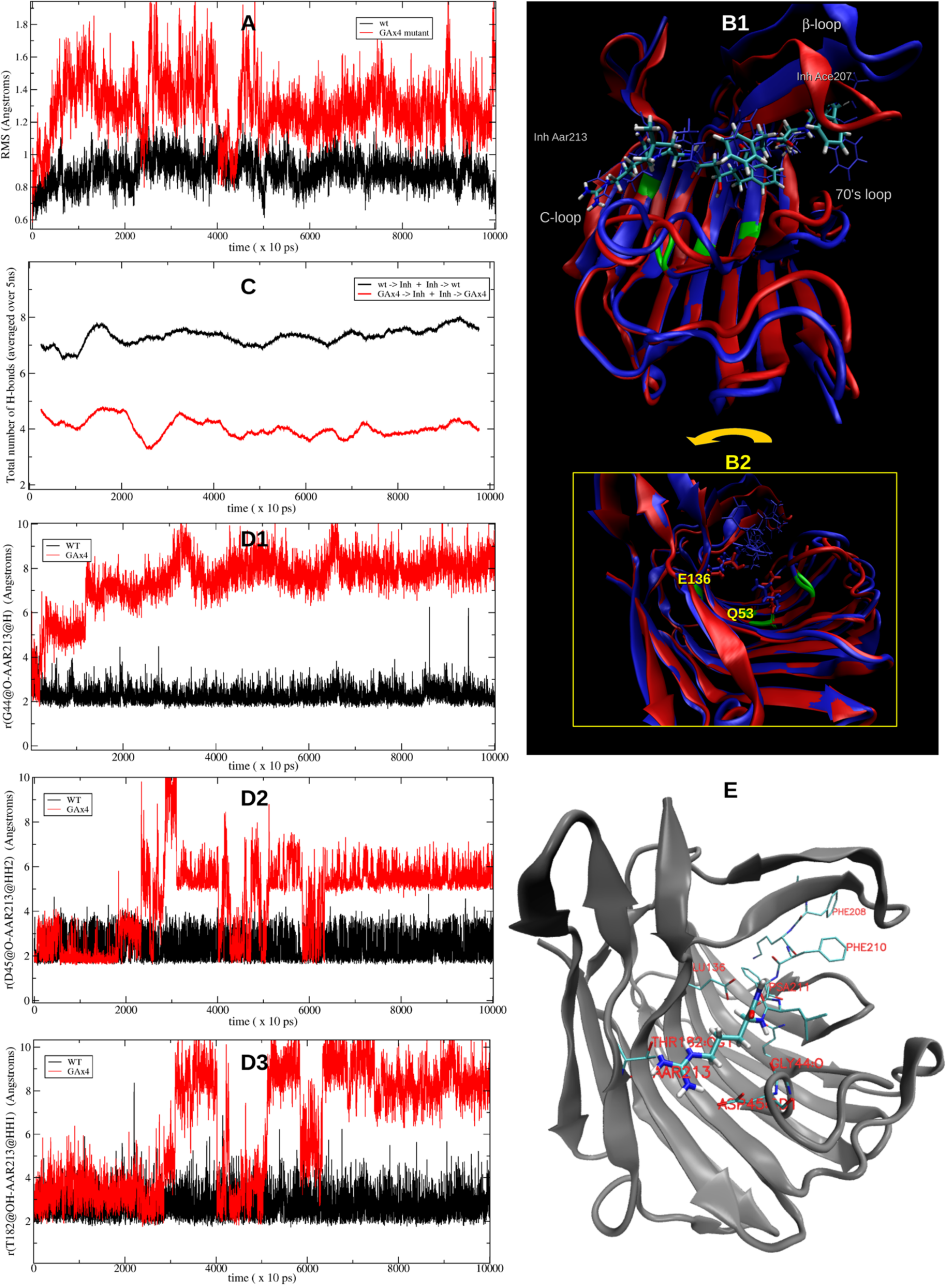
Molecular Dynamics Analyses of G8A-G41A-G44A-G55A mutant. **a** Comparison of the backbone RMSD of the WT and the GAx4 mutant. **b1** Comparison of the initial (red) and intermediate (37.000 to 37.005 ns, in blue) structures of the GAx4 mutant. The inhibitor (Inh) represented in licorice. The mutated residues G8A-G41A-G44A-G55A in green (on the cartoon). **b2** Rotated view with the catalytic dyad indicated. **c** Number of H-bonds (smoothed as running average over 5 ns or 500 frames) as donor and as acceptor between the WT and the GAx4 mutant with the inhibitor along the dynamics. **d1** Depletion of the contacts to the inhibitor in the GAx4 mutant (red) with respect to the WT (black) directly due to the G44A substitution, the backbone O with the backbone H of the inhibitor residue AAR213 is weakened. **d2** G44A substitution causes that the salt bridge involving the contiguous D45 with AAR213 is weakened. **d3** G8A substitution causes a weakened H-bond involving T182 alcohol O with AAR213. **e** Cartoon of 2IFW showing interactions lost or weakened in the GAx4 mutant as shown in D1-D3

In order to analyze the roles of the four glycines we performed 100 ns molecular dynamics with the GAx4 mutant as compared to WT. Fig. 3a shows that the GAx4 substitution yielded a much higher backbone RMSD, similar results were obtained for the overall RMSD on the inhibitor. A view of the structure averaged over intermediate 5 ps of the simulation (Fig. 3b1) shows major changes in the 70’s and β-loops as well as a third loop that is stabilized by a disulfide bridge, hence referred to as the C-loop. The change in the β-loop conformation causes a loss of hydrophobic contacts with the transition state inhibitor. This can also be observed in Additional file 5, where snapshots at four different simulation times (2.9, 4.8. 40 and 100 ns) illustrate the early opening of the β-loop. In addition, the GAx4 mutant shows both a repositioning of the catalytic dyad (E136/Q53) (Fig. 3b2) and a depleted H-bond donor/acceptor capacity, as compared to the WT (Fig. 3c), the net loss being between 3 and 4 H-bond contacts on the average.

The above considerations reveal a change in structure and possibly functionality. In order to confirm these observations, the standard free energy of binding of the eqolisin/inhibitor complexes were calculated for the WT and the GAx4 mutant using two different models (GB and PB) on the last 35 ns of equilibrated trajectories shown in Fig. 3 (Table 1). The substitution of these glycines causes a depletion of about 33 and 34 kcal/mol (GB and PB models, respectively). Even though the other nine mutants will also be analyzed within 40 ns of total simulation, for the particular case of the wt (reference) and this mutant, additional 60 ns of simulation were performed and the energetic analysis gave equivalent results (energies in parenthesis on Table 1). Besides the analysis of the contacts lost and structural changes (Fig. 3 and Additional Files 6 and 7), an analysis of the essential modes of motion of the complex and the individual contacts was performed to rationalize such a high change in the affinity for the substrate for the mutant as well as for establishing the role of each of the four G to A mutation. The overall cross-correlation function of G41, G55 in their β-sheets and G44 (and the subsequent C-loop) shows they are correlated with the inhibitor (res # 207–213), the β-loop (res. #141–148), the 70’s loop as well as with Q53 and W67 (Data not shown), correlations that are diminished or lost in the GAx4 mutant. MD analysis of the four independent Gly to Ala mutants (details on Additional files 6 and 7) shows that the largest contribution to the loss in binding energy is derived from G55A (See Table 1). The correlations of their motions, some of which are rather long-ranged, can be rationalized by means of the contribution given by the first three lowest essential normal modes shown on Additional file 8. Two of the three first modes are remarkably different for GAx4 with respect to WT.

**Table 1:** Calculated free energies of binding

	*ΔG*^0^ binding / kcal/mol	
Protein/Inhibitor complex	PB model	GB model
wt	−78.74 (79.69)^a^	−93.27 (93.76)^a^
G8A-G41A-G44A-G55A	−45.10 (44.87)^a^	−60.24 (59.74)^a^
G8A	−76.31	−87.97
G41A	−70.50	−85.14
G44A	−73.16	−87.87
G55A	−57.50	−71.27
W67F	−69.73	−86.64
W67F-L105W	−83.39	−93.54
L105W	− 75.03	−89.68
P72K	−72.82	−86.89
D43A	−78.39	−88.34

^a^Values obtained from the analysis of the extended 100 ns trajectories

Two additional evident observations about the role of these glycines are revealed in the dynamics of the WT. G44 forms an H-bond to the AAR213@H residue of the inhibitor through its backbone O. The G55 is tightly H-bonded through their backbones with W67 and G44; the latter is at the beginning of the turn lead by D45, which is comprised in a tight salt bridge with the positively charged guanydonium group of the AAR213 residue of the inhibitor, also persistent during the whole simulation in the WT. On the other hand, G8 (which has also a high cross-correlation value with AAR213 and participates into the lowest essential mode of the WT) is H-bonded with the backbone of T182, which is also H-bonded to AAR213 through its alcohol oxygen during the whole simulation of the WT. The change in the flexibility of the backbone of G8 and G44 by the alanines in the mutant affects both directly and indirectly these three strong contacts: D45@Od – AAR213@Hh22 (because of A44), A44@O–AAR213@H (because of A44 itself) and Q182@Og – AAR213@Hh12 (because of A8). Fig. 3d and e illustrate the persistence of these contacts along the whole simulation for the WT and their depletion in the GAx4 mutant. Even though G55A substitution was found to maximally affect the energetics of binding, all the above important contacts were also found to be seriously depleted in the G8A, G41A and G44A individual mutants (further details in Additional files 6 and 7).

SNAP analysis using 2IFW as template shows that the identified substitutions of G41 and G44 are expected to have an effect on the enzyme’s functionality (Additional File 3). As a result of these evaluations, we considered G8, G41 and G44 might well be strictly conserved, which we included in the sequence scrutiny in order to obtain a specific dataset that lacks noise in the identification of SDPs.

### Co-evolution of W67F and L105W mutations resulted in higher binding energy to inhibitor

**Fig. 4:**
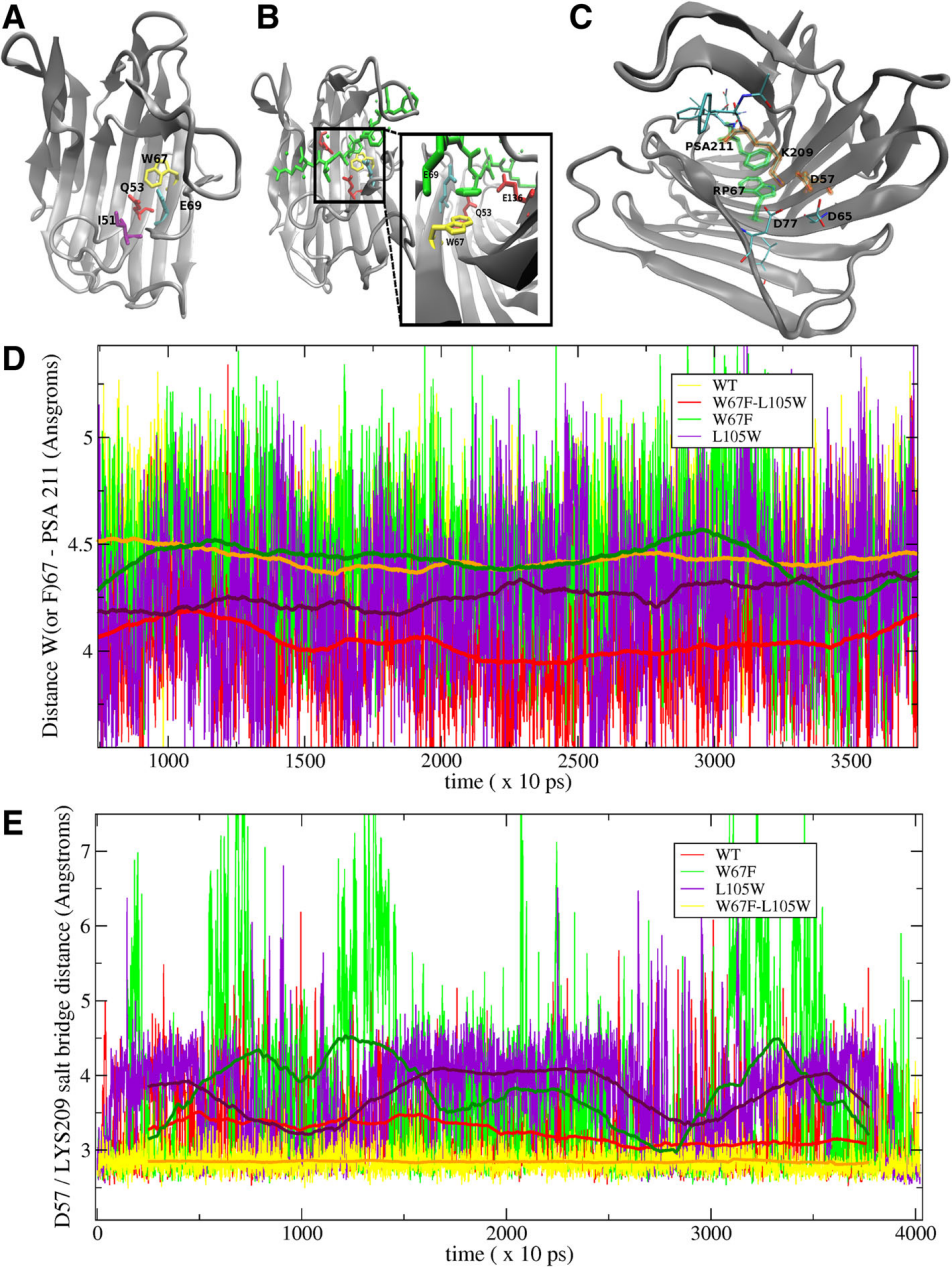
Structural and Molecular Dynamics of W67F and W67F-L105W mutants. **a** Cartoon of 2IFW showing I51 (purple) at the start of a β-sheet as well as W67 (yellow) and E69 (cyan) relative to catalytic Q53. **b** Cartoon of 2IFW detailing the π–π stack between W67 (yellow) and PSA211 from the transition state inhibitor. **c** Cartoon of 2IFW showing π–π stacking (green licorice) and salt bridge (orange licorice) between eqolisin and transition state inhibitor. **d** Distance and its running average (over 5 ns, smoother solid line) for the π–π stacking of residue 67 with the inhibitor PSA211 phenyl ring in the WT, the W67F and L105W mutants as well as the W67F-L105W double mutant as determined by molecular dynamics simulation **(e)** Distance between D57@Og and LYS209@Hz in the WT, the W67F and L105W mutants as well as the W67F-L105W double mutant as determined by molecular dynamics simulation

Another highly conserved residue is W67. Fig. 4a shows that the bulky and rigid side-chain of W67 is involved in positioning Q53, assisted by the strictly conserved E69. Furthermore, its indole aromatic ring forms a π–π stacking with the PSA211 from the transition state inhibitor that was crystallized with SCP-B (See Fig. 4b). This indicates a role in substrate binding. A total of 12 sequences show substitution W67F. Albeit smaller, a phenylalanine can still be envisaged to fulfill both hypothesized functions. Interestingly, five of 12 sequences, although from a single clade, also show W105 which is an otherwise highly conserved small hydrophobic residue (L, I, V or M) in the outer pleated β-sheet directly below position 67. The more bulky W105 can be envisaged to change the conformation of the inner pleated β-sheet such that the aromatic ring of the F67 occurs at the same position as the typical W67, thereby sustaining for the π–π stacked stabilization of the substrate. In order to test this hypothesis and isolate the main structural factors which could rationalize it, models of the W67F, L105W and the double mutant (W67F/L105W) were simulated by means of molecular dynamics and compared to the WT structure. Mutant W67F shows a similar RMSD as the WT whereas L105W shows a slightly higher RMSD. The double mutant W67F/L105W showed a slightly reduced RMSD. Similar results were obtained for total inhibitor RMSD (Additional file 9). The π–π stack distance of mutant W67F shows a persistence in time as the WT, L105W shows a slightly higher distance whereas double mutant W67F/L105W showed a slightly tighter interaction (Fig. 4c and d). Additional noticeable differences arose, especially in the case of L105W, which was unable to keep residue 209 of the inhibitor tightly bound during the whole simulation. Subtle differences, with impact in the overall energy balance, also appeared for W67F. L105W and W67F show about 3 and 6 kcal/mol, respectively (similar for PB and GB models See Table 1), less affinity for the substrate analog than the WT. On the other hand, the double mutant yielded a free energy of binding more favored than the WT (4 and 0.3 kcal/mol more negative than the WT for PB and GB, respectively). This suggests the bulkier W105 pushes F67 up to the aromatic moiety of the inhibitor, thus leading to a better π–π interaction than found for W67/L105 in the WT.

Besides its direct interaction with PSA211, WT dynamics demonstrates other important interactions. The squared cross-correlation function of W67 shows an important peak with PSA211, but it also shows W67 is correlated with G55, D57, D65, D77, L105 and D136 (all of them > 0.55, Additional file 10). In the double mutant, the bulkier W105 not only pushes F67 up to the inhibitor, it also repositions D57, in the WT comprised in a salt bridge interaction with the LYS209 of the inhibitor. A stable H-bond network between LYS209, D65 and D77 also depends on the right position of D57 for a tight salt bridge. Fig. 4c and e show the persistence and closeness of this contact during the simulation. Indeed, the combined W67F and L105W substitutions were found to be a better combination for properly holding D57 tightly bridged to the inhibitor. This would contribute to the favored binding energy for the double mutant. Besides its small improvement in the binding energy, it is worth to mention that the analysis of all the contacts (H-bonds, electrostatic and hydrophobic) from the double mutant closely resembles the analysis for the WT. Also the first essential modes are very similar to the WT as shown on the Additional file 8. Thus whilst, either W67F or L105W substitution have little effect or deplete the binding modes and energy, the double mutant should equal or even improve the WT ability for binding this substrate/ transition state analogue. As a result of these evaluations, we consider both the W67F and the L105W as functional substitutions that seem to have co-evolved.

### The P to K substitution at position 72 depletes inhibitor affinity

P72 is part of the 70’s loop, described by Pillai and collaborators [15] and six sequences in the initial set of sequences have a substitution in that position. First the aforementioned EFBso00001 has a serine. Then, EFPde00001 (Fig. 2), EFMac00003, EFMan00003, and EFCm000003 have a lysine whereas EFCm000002 has a glutamine. The P72K substitution was modeled and a structural alignment (data not shown) shows a structural change in the β-loop. These sequences occur at relatively large distances in a preliminary tree, which suggests the sequences might encode NFHs.

In order to test the hypothesis that P72K disrupts function, we performed molecular dynamics simulations with the P72K mutant. The structure is, in general, well conserved during the simulation (see Additional file 11 A, showing the backbone RMSD compared to the WT). The substitution has an overall effect less evident than in the case of W67F and fairly smaller than GAx4 and D43A. Most contacts and the overall mode of binding to the inhibitor is similar to either the WT and the W67F-L105W. However the mobility of the 70’s loop is affected and additional interactions involving the ammonium group of K62 and alternatively N144@O N144@Od appeared, thus altering the essential mode involving the 70’s- and β-loops (see Additional file 11 B); all these interactions were absent in the WT, having the hydrophobic and compact proline residue. The substitution P72K thus depleted the affinity of the mutant for the inhibitor by about 6 kcal/mol in both models for computing the ***Δ****G*^0^ of binding. As a result of these evaluations, we consider that the P72K mutation possibly disrupts function and sequences were removed accordingly. Since the P72S mutant also contains Q51 (see below) at a strict small hydrophobic site and there was no indication for another co-evolution event this was also considered a problematic sequence. Then if we consider that these mutations likely disrupt function, the same can be argued for the remaining P72Q. All the mentioned substitutions were predicted by SNAP (Additional file 3) as non-neutral and corresponding sequences were removed.

### An additional check about the importance of D43

The importance of D43 for the eqolisin function was raised by other authors [8] and although it was not the focus of the present study, we checked the behavior of the D43A mutant in order to have an additional check on the reliability of the MD analysis done for all the in silico mutants discussed here. Indeed, the D43A substitution depleted the ability of the protein for binding the TS analog inhibitor. In Additional file 12 b it is shown how the ß-loop opens and looses contacts with the inhibitor. This resembles the problem observed in the case of the GAx4 mutant (Additional file. 5). Despite that the loss in binding energy is less remarkable than in the case of the GAx4 mutant (Table 1), the overall RMSD is much higher, especially the RMSD of the inhibitor in the site, as shown compared to the WT in Additional file 5a. In summary, D43A was found to be disruptive, using the same criteria applied to the other mutants analyzed here.

**Fig. 5:**
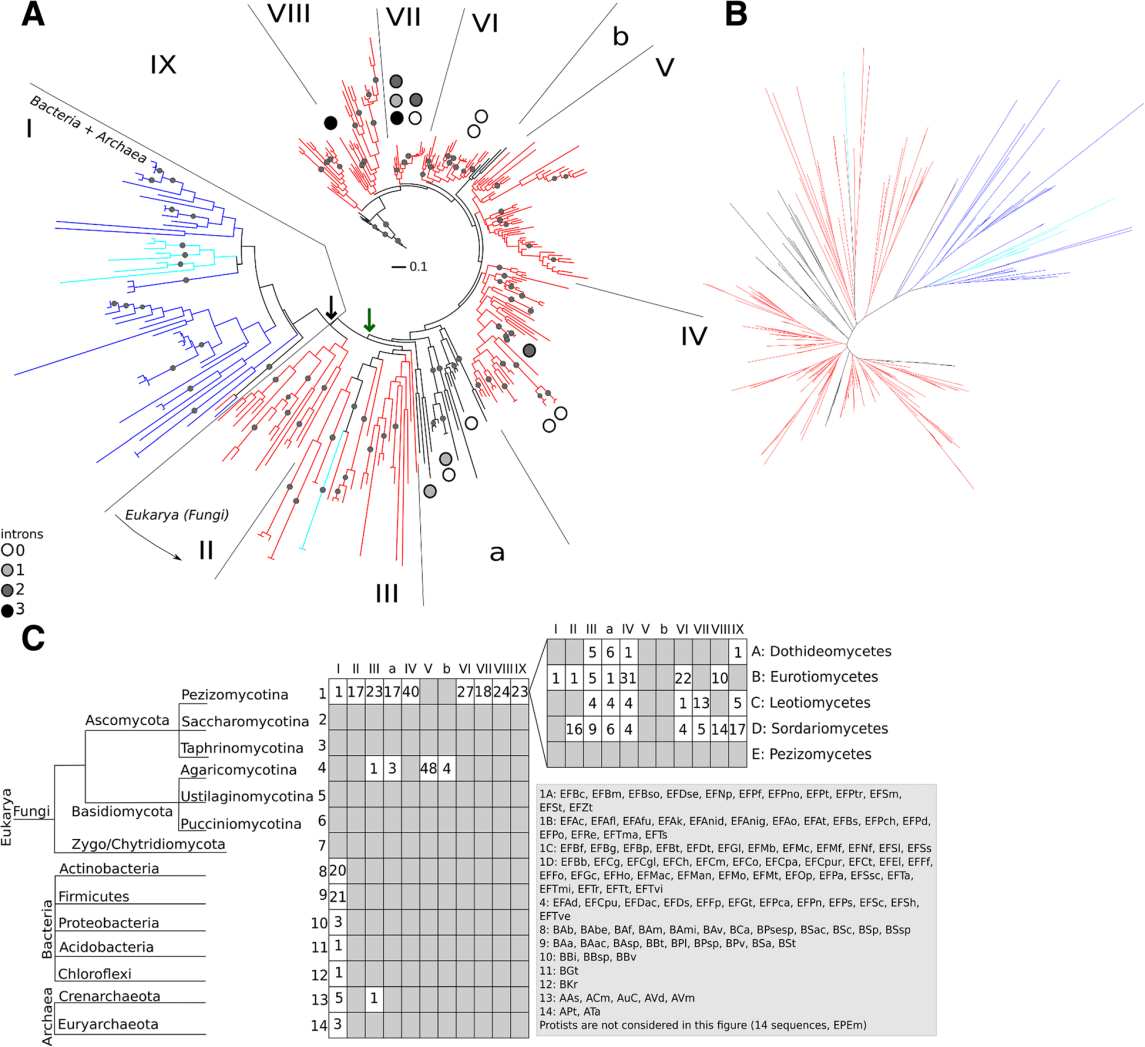
Phylogenetic Analysis of Eqolisins. **a** Radial phylogram. Fungal sequences are in red or black (small clusters indicated with a and b), Bacterial sequence in blue and Archaeal in cyan. The black and green arrows indicate two possible common ancestors discussed in the text. Roman numerals refer to selected clades discussed in the main text. Dots indicate Bayesian support > 0.8, scale bar represents a distance of 0.1 accepted amino acid mutations per site. **b** Radial cladogram. **c** Taxonomic distribution of eqolisins on a tree representative for generally accepted consensus species phylogenies. Roman numerals and a and b refer to the selected clades (Fig. 5a). Numbers indicate the number of sequences per clade and subphylum/class. Abbreviations in Additional file 15

### Additional low frequency substitutions

A number of sequences have substitutions at position D43, which forms a minor turn in 2IFW (not shown) and has been shown to be important for function [8]. Three sequences, one bacterial and two eukaryotic, have S, which, when phosphorylated, is physicochemically similar to aspartic acid [43], hence these are envisaged non-fatal substitutions. Ten sequences have G43, a relatively large number that, combined with its appearance in fungi, bacteria and archaea, suggests this is another acceptable substitution. Furthermore G is often found at turns and can be envisaged to maintain functionality.

Position 51 has a highly conserved small hydrophobic residue, L, I or V, where EFTa000006 has P instead. The isoleucine in 2IFW occurs at the start of an inner sheet element and has its side-chain pointing inward the halfpipe (Fig. 3a). It seems unlikely that substitution by the rigid proline would not affect catalytic efficiency, hence we consider EFTa000006 (Fig. 2) as an NFH. EFBso00001 has Q51 (not shown) as well as the aforementioned P72Q mutation and since there are no indications for co-evolution this sequence also probably encodes an NFH. SNAP analysis (Additional file 3) predicts both substitution have an effect on function.

Three bacterial sequences have a phenylalanine at position 89 that is otherwise characterized by VILM. Since phenylalanine is hydrophobic, this appears as an allowed substitution. Position 105 is another conserved hydrophobic site, typically constituted by VILM but shows, besides the above discussed Ws, two instances of phenylalanine, one of tyrosine and one of glutamate. The glutamate substitution in BCa0000005 would result in a buried but charged side-chain, which could be explained by a co-evolved basic residue, which could not be identified. Hence, this is probably a substitution that disrupts function. The tyrosine substitution in APf0000001 might well be a problem of erroneous sequencing or gene modeling since it is surrounded by a stretch of about 20 to 40 amino acids that poorly align and the sequence was removed. The two instances of phenylalanine substitutions in bacterial (Bgt0000001) and eukaryotic EFCm000005 introduce another hydrophobic but larger residue and given the partial solvent exposure, it can be envisaged this substitution is not fatal. Finally, the highly conserved site 133 has VILM and two, likely partial sequences (EFFo000002 and EFNh000001) showing a gap in the MSA, these were also removed.

Upon removal of the problematic sequences we realigned the remaining sequences and checked the effect of adding the removed sequences to the alignment. TCS confirmed the addition of the sequences resulted in a score drop of 800 to 773, confirming the sequence scrutiny resulted in a more reliable MSA. Finally, an additional 23 scrutinized novel eqolisin sequences were added.

### Phylogenetic clustering suggests fungal Eqolisins have resulted from an ancient LGT event

**Fig. 6:**
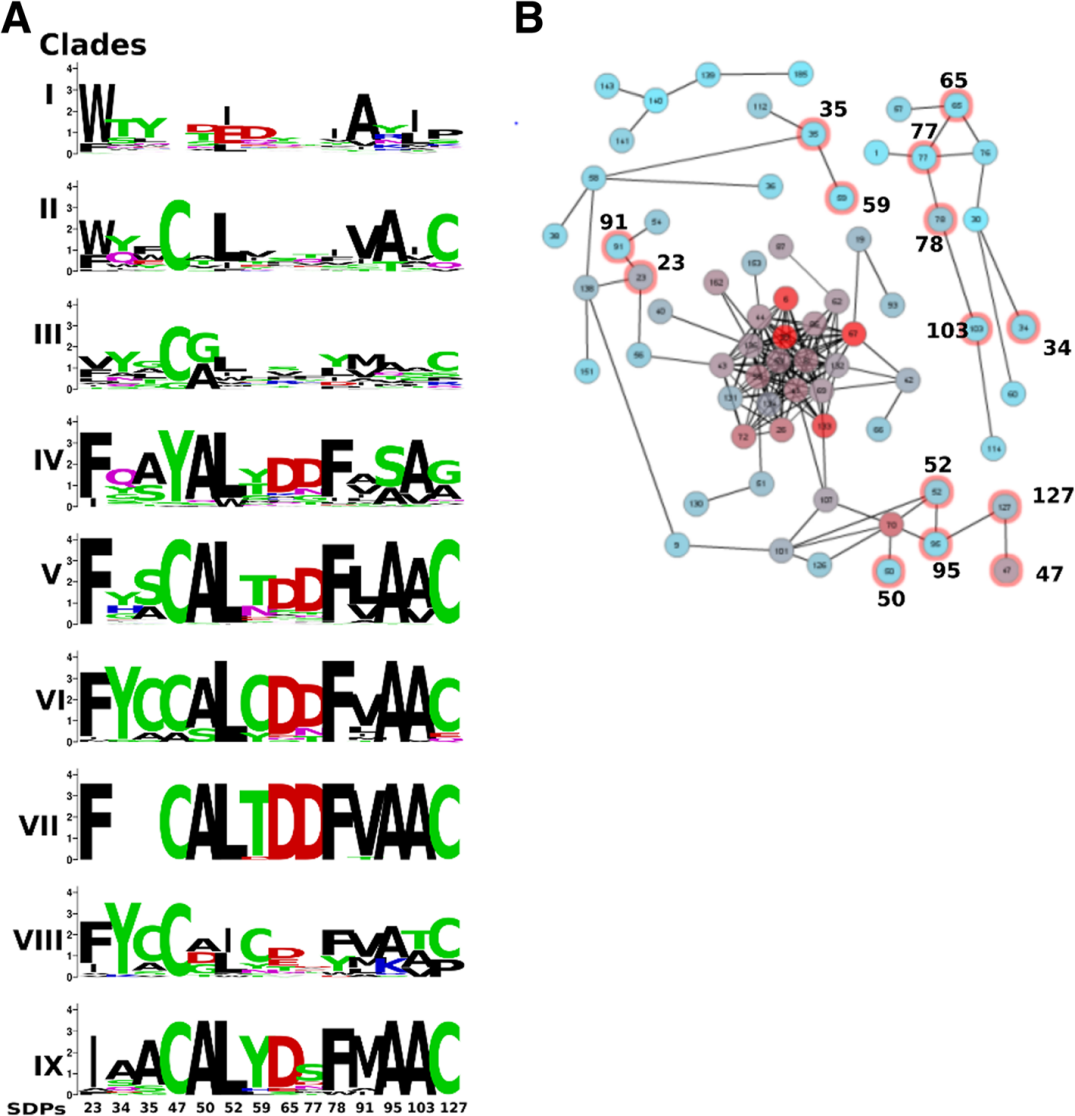
Specificity Determining Positions of Eqolisins. **a** Sequence logos of SDPs per selected clade (Fig. 5). **b** Mutual Information Network. Connected nodes have MI of at least 6.5. Note that a first SDN (SDPs 47, 52, 95, 127 and 50) is connected to the major network, whereas the other SDN (65, 77, 78 and 103) is not. Color of the nodes corresponds to Kullback Leibler conservation

A new MSA of a total of 314 sequences was made (Additional file 13), subjected to trimming of poorly aligned subsequences and used for phylogeny. First a maximum-likelihood tree was constructed, which was then used to initiate a Bayesian analysis. Fig. 5a shows an annotated radial phylogram, a radial cladogram is shown in Fig. 5b. Cluster assignation was made based on monophyly and, more importantly, taxonomical considerations. Apart from two orphan sequences, prokaryotic (archaeal and bacterial) and fungal sequences cluster separately which suggests a common ancestor, separating prokaryotic clades I and II from the fungal sequences. However, a taxonomical distribution analysis (Fig. 5c) points towards an alternative common ancestor, also indicated in Fig. 5a. The majority of the sequences are found in the subphylum pezizomycotina, which is further classified in the classes of dothideomycetes, eurotiomycetes, leotiomycetes, sordariomycetes and pezizomycetes, the last class not containing eqolisins. Clade III is a monophyletic clade with sequences of all four classes and since it is closest to the initially suggested common ancestor, this indicates the common ancestor might correspond better with the node between the clade containing subclades I, II and III and the rest of the tree. This is substantiated by the sequence logo analysis (Fig. 6a) presented in the description of the structure-function analysis. All other assigned clades were selected based on monophyly and size (a requirement of at least 10 members was set arbitrarily).

Lateral gene transfer (LGT) seems to have had an important role in the eqolisin phylogeny. The two orphan sequences are examples of LGT. Furthermore, the lack of eqolisin encoding sequences in eukaryotes other than fungi points towards an ancestral LGT event. The taxonomical distribution of sequences suggests this ancestral LGT has taken place between a bacterium and the ancestor of the Dikarya. The absence of introns in many, albeit not all fungal sequences corresponds with the proposed ancestral LGT.

### Functional diversification of substrate binding site

Since a number of fungi have various paralogs in well separated clades, fungal eqolisins might have been subject to functional diversification. We performed analyses in order to identify first Cluster Determining Positions (CDPs) and then Specificity Determining Positions (SDPs). CDPs are positions in the protein (or columns in the corresponding MSA) that significantly contribute to clustering. These are the result of either genetic drift (i.e. neutral substitutions) or selection, which would mean they are somehow related to functional or structural diversification. Functionally, but also structurally, important residues are likely to show moderate to high levels of interaction with other residues, which can be determined with the measure of mutual information (MI). Hence, CDPs that show high MI with other residues are likely important in either maintaining the structure or functional diversification. Since MI reflects co-variation, groups of CDPs and other positions that are directly connected via significant MI values are expected to affect the same functional aspect.

CDPs were identified using SDPfox [44], using the clustering indicated in Fig. 5. Out of 28 CDPs that were identified, 20 showed significant cumulative MI levels, as determined by Mistic [45], of which a total of 14 could be confirmed by H2Rs [46], all considered putative SDPs (pSDPs). A resume of the SDP identification is shown in Additional file 14 that also contains a description of the binding cleft, based on the work of Pillai and co-workers on SCP-B [15]. Sequence logos of the SDPs, according to the clustering shown in the phylogeny of Fig. 5., are shown in Fig. 6a alongside the MI network, defined as the subnetwork of nodes that directly connect to at least one other node with significant MI, obtained by Mistic (Fig. 6b). The MI network consists of three fully connected subnetworks. The large connected subnetwork contains an intricate central module of nodes, mostly corresponding to highly conserved sites, with some branches with lower levels of connections. This central module conceptually corresponds with the core eqolisin function and lacks SDPs. Two instances of paired SDPs, (35 and 59; and 23 and 91 respectively) are found in one of the branches with low connection levels and appear to be the result of co-evolution driven by of structural compensation. The sequence logo shows SDP35 and 59 have a preferred C in both clade VI and VIII that, combined with their proximal three dimensional location, points toward a possible disulfide bridge. SDPs 23 and 91 are likely involved in an important hydrophobic interaction given the substitution pattern shown in Fig. 6a and their 3 dimensional proximity in the protein (not shown). These pSDPs are as such not considered real SDPs since we cannot foresee any functional diversification. A first specificity determining network (SDN) with four directly connected SDPs (47, 52, 95 and 127) as well as SDP50 that connects via single non-SDP to SDP52 and SDP95, is found in one of the branches with lower levels of connections. A second SDN with four directly connected SDPs 65, 77, 78 and 103 locates into one of the small (*n* = 11) subnetworks that also includes pSDP34. The second small (*n* = 5) subnetwork contains no SDPs.

**Fig. 7:**
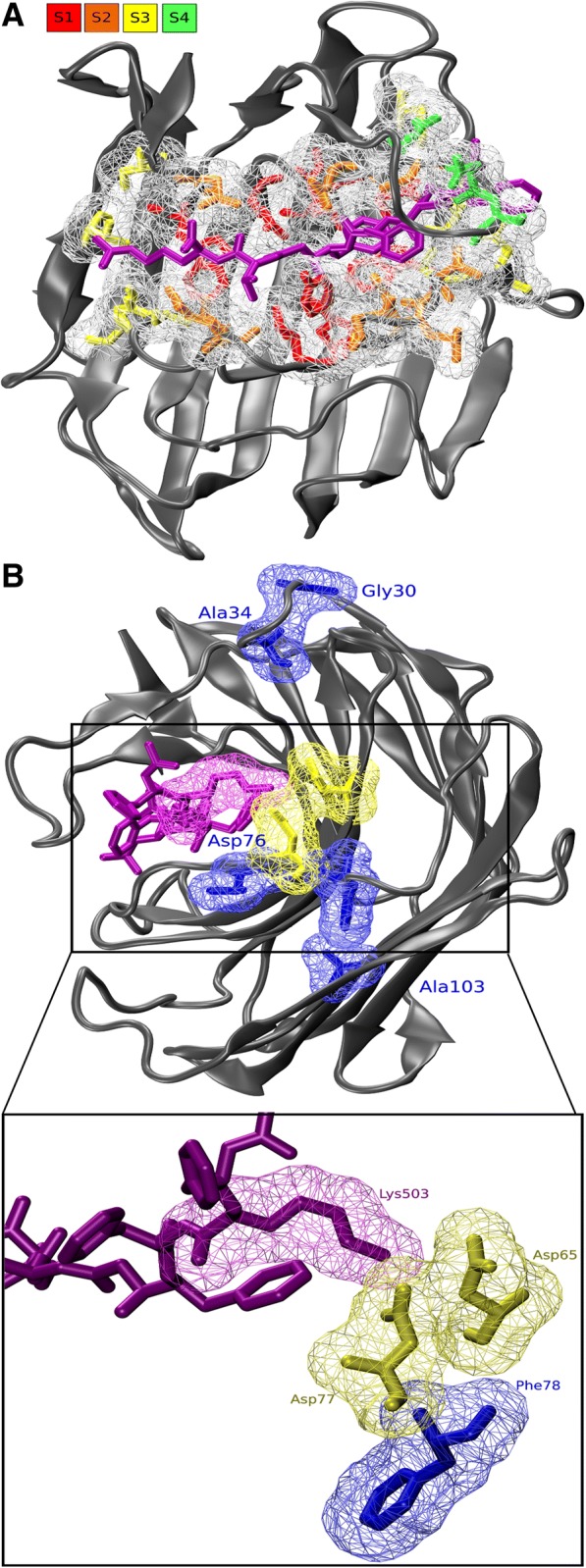
The D65-D77 Mutual Information network is involved in substrate specificity. **a** Cartoon of 2IFW with highlighted binding cleft. Transition state inhibitor is indicated in purple licorice. Sites are indicated in colored licorice (see legend) with surface indication. **b** Interaction of D65 and D77 with substrate analog. The inset shows that the acid groups of both aspartates (S3’ yellow surf) interact directly with the basic group of the Lysine form the the analog (purple). Other SDPs of the same SDN are indicated in blue (See also Additional file 16)

The SDN in the small subnetwork is dominated by the preferred D65, D77 and F78 in groups IV to IX (Fig. 6a). Fig. 7 shows how D65 and D77, part of subsite S3, interact with the substrate. This is further substantiated by MD analysis. Squared cross correlation function of D65 shows high levels of interaction with for instance D77 and the Lys in the substrate (See Additional file 11). Both acid side chains interact directly with the basic sidechain of the Lys in the substrate analogue. F78 does not form part of the binding cleft, but interacts directly with its neighbor D77. A103 connects to F78, having an MI value of 6.7 and Fig. 7b shows they interact physically. Hence, SDPs 65 and 77 are predicted to affect substrate specificity, particularly concerning P3. Positions 78, 103 likely give some sort of structural support. The information contents at positions 65 and 77 in the various clades is likely indicative for substrate specificity.

**Fig. 8:**
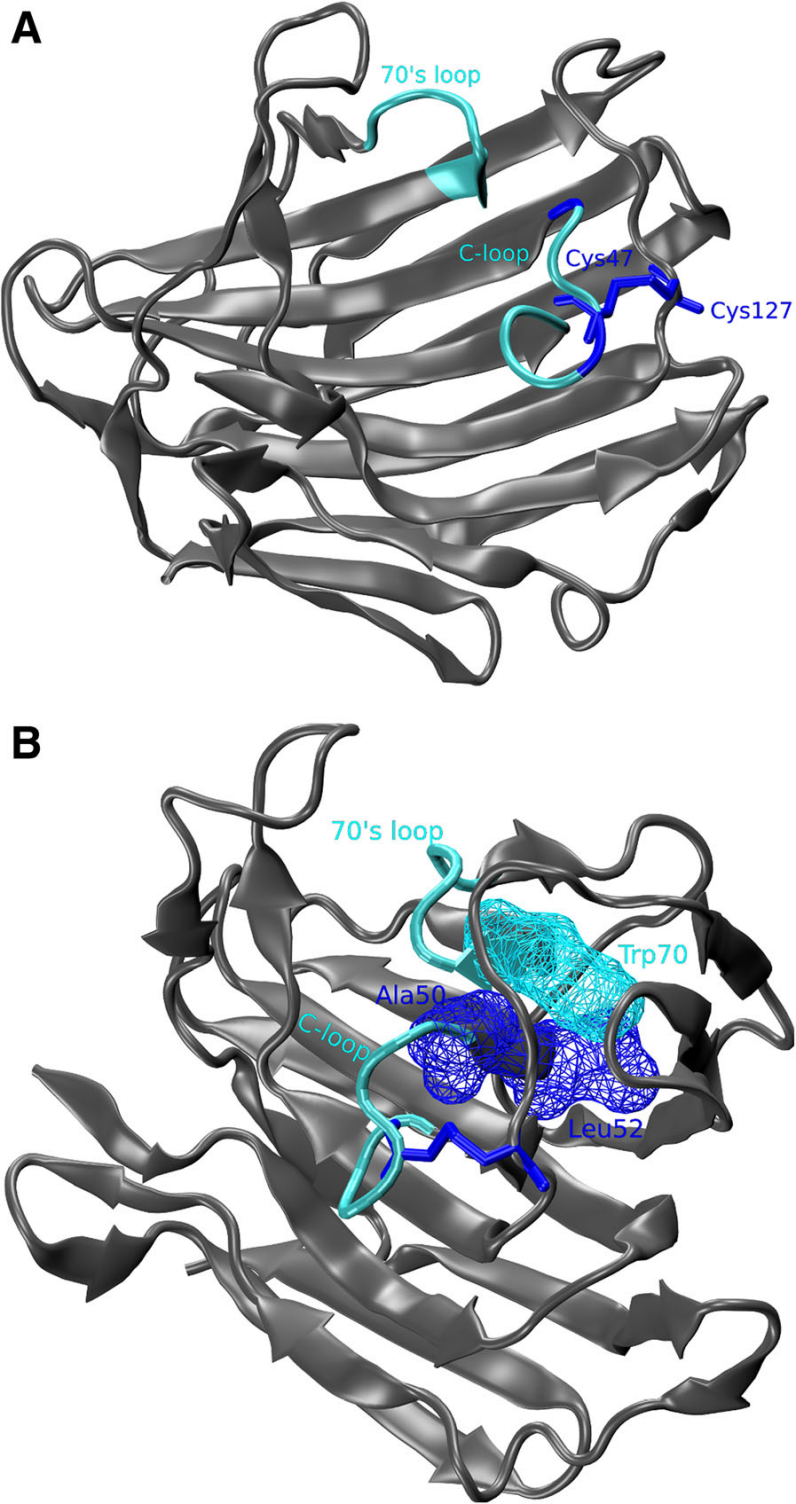
The C-loop interacts with the previously described 70s-loop. **a** Cartoon of 2IFW with highlighted the previously described 70s-loop and the novel C-loop (Cyan). C47 and C127 (blue licorice) form a disulfide bridge that holds the loop in place. The C-loop also includes SDP50 (blue). **b** Cartoon of 2IFW with highlighted the 70s- and C-loop. Y70 interacts physically with A50 and L52, both identified as SDPs

The SDP group in the large subnetwork contains SDPs dominated by the Cysteins at 47 and 127 that form a disulfide bridge in SCP-B and which are present in most sequences, except for those in clades I and IV (Fig. 6a). In SCP-B the disulfide bridge stabilizes a hitherto un-described loop from position 44 to 51 which we refer to as the C-loop. Fig. 8a shows the C-loop, the disulfide bridge as well as the 70s loop since the central position in group 2 is taken by position 70, a Tyr in SCP-B. This suggests the C-loop interacts with the 70s-loop, which has been shown to be important in binding dynamics. Fig. 8b demonstrates that in SCP-B both SDPs A50 and L52 interact physically with Y70, likely influencing the mutual positioning of the 70s- and C-loops. Clade IV sequences have Y47, which appears to be compensated by the substitution C127A. SDP127 is connected with SDP95, which is encountered in the outer sheet just below the inner sheet and the C-loop. This suggests a secondary, structural compensation but the substitution pattern (A95S) does not give any clue on how this might be established. The C-loop contains four residues that are part of the binding cleft (Additional file 11) of which, interestingly 51 is another CDP that does not form part of the network. All together this suggests that the C-loop is involved in the dynamics that lead to substrate binding.

## Conclusions

Only few eqolisins exist but interestingly fungi that secrete acid as part of their lifestyle can have up to nine paralogs, which implies a process of functional redundancy and diversification has occurred. This was studied using a stringently mined sequence set. A number of sequences we removed might be functional but we prefer to prevent possible contamination with sequences of NFHs. An interesting case of recent molecular evolution was identified, that given a more favorable binding energy towards the transition-state inhibitor, most likely resulted in a more active enzyme. Furthermore we detected two groups of SDPs, one that very likely is involved in substrate specificity given two of the SDPs form part of the binding cleft. The other group seems to affect loop dynamics. Some of the additionally identified CDPs might also be SDPs but the lack of MI signal cannot corroborate them.

## Additional files

Additional file 1: List of the protomers tested (i.e. those with complete 40 ns simulations) showing the averaged RMSD. ASH and GLH are aspartic and glutamic acid, respectively, in protonated form (RCOOH neutral form); acidic residues not listed are anionic (RCOO-). All arginines and lysines protonated; no histidines are present in the 2IFW sequence. (DOC 87 kb)

Additional file 2: Complete Preliminary Multiple Sequence Alignment. (FAA 354 kb)

Additional file 3: Table resuming TCS and SNAP analyses. Each of the removed sequences was tested by TCS. Consensus score was 79 and the lowest scoring accepted score was 51. All dubious mutations were analyzed by means of SNAP using 2IFW as a template. E indicates the prediction is that the mutation has an effect on enzyme activity, N means the mutation is predicted to be neutral. NA: Not applicable. (DOC 41 kb)

Additional file 4: Molecular Dynamics Simulation determining best protomer state. **(A)** Backbone RMSD of the best protomer for the WT compared to five of the other best. **(B)** Backbone RMSD of substrate analog inhibitor for the best protomer for the WT compared to five of the other best. (TIF 830 kb)

Additional file 5: **(B)** Cartoons of the GAx4 mutant at 2.9, 2.4, 40 and 100 ns of simulation showing displacement of the β-loop relative to the inhibitor (solid golden surface). (TIF 3478 kb)

Additional file 6: Analyses of the changes in contacts for each individual GxA mutant, as compared to the WT. **(A)** The salt bridge D45-AAR213 is weakened for G41A and G55A and depleted for G8 and G55 (as in the case of GAx4). **(B)** The H-bond to AAR213 from the backbone G41 (or A41) oxygen is lost for G41A and weakened in the other three single mutants. **(C)** The H-bond involving the T182@OG is weaker than in the WT for the case of G41A and depleted or lost for G8, G44 and G55. Smooth thick lines are 200 ps running averages. (TIF 4486 kb)

Additional file 7: Analyses of the changes in contacts for each individual GxA mutant compared to either WT or W67F-L105 double mutant. **(A)** The salt bridge between D57 and LYS209 of the inhibitor (which is tightest in the case of W67F-L105W) is depleted in the four GxA substitutions, especially for G8A and G55A. **(B)** Also the π-π stacking with PSA211 results weaker in the individual mutants G8A and G41A and practically absent in G55A. Smooth thick lines are 200 ps running averages. (TIF 3253 kb)

Additional file 8: (**A1-A3)** Ca vectors of the first essential normal mode from PCA analysis for the GAx4, WT and W67F-L105W species. **(A4)** the squared displacement of each residue in the first mode. B-C squared displacement of each residue on the next two modes. (TIF 1669 kb)

Additional file 9: RMSD of the inhibitor residues for the WT, W67F, L105W and the double mutant. (TIF 2432 kb)

Additional file 10: Squared cross correlation function of W67 against all other residues (wt trajectory). (TIF 1550 kb)

Additional file 11: Backbone RMSD comparison for the WT and the P72K mutant. (TIF 2162 kb)

Additional file 12: Molecular Dynamics Analyses of D43A mutant. **(A)** Comparison of the backbone RMSD of the WT and the D43A mutant. **(B)** Cartoons of WT and D43A mutant showing displacement of the ß-loop relative to the inhibitor (solid golden surface). (TIF 397 kb)

Additional file 13: Complete Final Sequence Alignment. (FAA 379 kb)

Additional file 14: Resume of the SDP identification by crossing SDPfox and Mistic analysis. (XLS 21 kb)

Additional file 15: Abbreviations of species and their taxonomical distribution. (XLS 58 kb)

Additional file 16: Squared cross correlation function of D65 against all other residues (WT trajectory), showing main peaks at D57 and D77 as well as inhibitors LYS209, which are involved in a network of H-bond and salt bridges network. (TIF 1361 kb)

## Abbreviations

GB: Generalized Born
LGT: Lateral Gene Transfer
MD: Molecular Dynamics
MI: Mutual Information MSA
MSA: Multiple Sequence Aignments
NFH: Non-Functional Homolog
PB: Poisson-Boltzman
PME: Particle Mesh Ewald
RMSD: Root Mean Squared Deviation
SDN: Specificity Determining Network
SPD: Specificity Determining Positions
WT: Wild type
