# Supplementary material for: Molecular dynamics and structure function analysis show that substrate binding and specificity are major forces in the functional diversification of Eqolisins
Source: BMC Bioinformatics. 2018 Sep 24;19:338. doi: 10.1186/s12859-018-2348-2 (PMC6154417; doi:10.1186/s12859-018-2348-2)
Supplement: Supplementary file 1 — List of the protomers tested (i.e. those with complete 40 ns simulations) showing the averaged RMSD. ASH and GLH are aspartic and glutamic acid, respectively, in protonated form (RCOOH neutral form); acidic residues not listed are anionic (RCOO-). All arginines and lysines protonated; no histidines are present in the 2IFW sequence. (DOC 87 kb) [file 12859_2018_2348_MOESM1_ESM.doc]

| **Label/RMSD (Å)** | **List of protonated acid residues (Residue-H/Position)** | | | | | | | | | | | | | | | | | | | | | | | | | | |
| --- | --- | --- | --- | --- | --- | --- | --- | --- | --- | --- | --- | --- | --- | --- | --- | --- | --- | --- | --- | --- | --- | --- | --- | --- | --- | --- | --- |
| **wt12c** | GLH | ASH | ASH | ASH | ASH | GLH | GLH | ASH | GLH | ASH | ASH | GLH | GLH | GLH | ASH | GLH | GLH | ASH | GLH | GLH | ASH | ASH | GLH | ASH | ASH | ASH | ASH |
| **0,89** | 3 | 15 | 43 | 61 | 65 | 69 | 73 | 76 | 85 | 87 | 98 | 121 | 132 | 136 | 137 | 139 | 140 | 147 | 149 | 161 | 164 | 170 | 172 | 177 | 178 | 190 | 193 |
| **wt0k** | GLH | ASH | ASH | ASH | ASH | GLH | ASH | GLH | ASH | ASH | GLH | GLH | GLH | ASH | GLH | GLH | ASH | GLH | GLH | ASH | ASH | GLH | ASH | ASH | ASH | ASH |  |
| **1,26** | 3 | 15 | 43 | 61 | 65 | 69 | 76 | 85 | 87 | 98 | 121 | 132 | 136 | 137 | 139 | 140 | 147 | 149 | 161 | 164 | 170 | 172 | 177 | 178 | 190 | 193 |  |
| **wt1** | GLH | GLH | GLH | ASH | GLH | ASH | ASH | GLH | GLH | ASH | ASH | GLH | ASH |  |  |  |  |  |  |  |  |  |  |  |  |  |  |
| **1,29** | 69 | 73 | 85 | 87 | 136 | 137 | 147 | 149 | 161 | 164 | 170 | 172 | 193 |  |  |  |  |  |  |  |  |  |  |  |  |  |  |
| **wt10c** | ASH | ASH | GLH | ASH | GLH | GLH | ASH | ASH | GLH | GLH | ASH | ASH | GLH | ASH | ASH | ASH | ASH |  |  |  |  |  |  |  |  |  |  |
| **1,92** | 61 | 65 | 73 | 76 | 85 | 136 | 137 | 147 | 149 | 161 | 164 | 170 | 172 | 177 | 178 | 190 | 193 |  |  |  |  |  |  |  |  |  |  |
| **wt11c** | GLH | ASH | ASH | ASH | ASH | GLH | ASH | ASH | GLH | ASH | ASH | GLH | GLH | GLH | ASH | GLH | GLH | ASH | GLH | GLH | ASH | ASH | GLH | ASH | ASH | ASH | ASH |
| **1,69** | 3 | 15 | 43 | 61 | 65 | 73 | 76 | 77 | 85 | 87 | 98 | 121 | 132 | 136 | 137 | 139 | 140 | 147 | 149 | 161 | 164 | 170 | 172 | 177 | 178 | 190 | 193 |
| **wt1212c** | ASH | ASH | ASH | ASH | ASH | GLH | GLH | ASH | GLH | ASH | ASH | GLH | GLH | GLH | ASH | GLH | GLH | ASH | GLH | GLH | ASH | ASH | GLH | ASH | ASH | ASH | ASH |
| **0,97** | 15 | 43 | 57 | 61 | 65 | 69 | 73 | 76 | 85 | 87 | 98 | 121 | 132 | 136 | 137 | 139 | 140 | 147 | 149 | 161 | 164 | 170 | 172 | 177 | 178 | 190 | 193 |
| **wt2** | GLH | GLH | ASH | GLH | ASH | ASH | GLH | GLH | ASH | GLH | ASH | ASH | ASH |  |  |  |  |  |  |  |  |  |  |  |  |  |  |
| **1,49** | 69 | 85 | 87 | 136 | 137 | 147 | 149 | 161 | 164 | 172 | 177 | 178 | 193 |  |  |  |  |  |  |  |  |  |  |  |  |  |  |
| **wt3** | GLH | GLH | ASH | ASH | ASH | GLH | GLH | ASH | GLH | ASH | ASH | ASH |  |  |  |  |  |  |  |  |  |  |  |  |  |  |  |
| **1,34** | 69 | 85 | 87 | 137 | 147 | 149 | 161 | 164 | 172 | 177 | 178 | 193 |  |  |  |  |  |  |  |  |  |  |  |  |  |  |  |
| **wt4444c** | GLH | ASH | ASH | ASH | ASH | GLH | ASH | GLH | ASH | ASH | GLH | GLH | ASH | GLH | ASH | GLH | GLH | ASH | ASH | GLH | ASH | ASH | ASH | ASH |  |  |  |
| **1,23** | 3 | 43 | 57 | 61 | 65 | 73 | 76 | 85 | 87 | 98 | 132 | 136 | 137 | 139 | 147 | 149 | 161 | 164 | 170 | 172 | 177 | 178 | 190 | 193 |  |  |  |
| **wt444c** | ASH | ASH | ASH | GLH | ASH | GLH | ASH | ASH | GLH | GLH | ASH | ASH | GLH | ASH | ASH | ASH | ASH |  |  |  |  |  |  |  |  |  |  |
| **1,88** | 57 | 65 | 76 | 85 | 87 | 136 | 137 | 147 | 149 | 161 | 164 | 170 | 172 | 177 | 178 | 190 | 193 |  |  |  |  |  |  |  |  |  |  |
| **wt44c** | ASH | ASH | GLH | ASH | GLH | ASH | ASH | GLH | GLH | ASH | ASH | GLH | ASH | ASH | ASH | ASH |  |  |  |  |  |  |  |  |  |  |  |
| **1,67** | 65 | 76 | 85 | 87 | 136 | 137 | 147 | 149 | 161 | 164 | 170 | 172 | 177 | 178 | 190 | 193 |  |  |  |  |  |  |  |  |  |  |  |
| **wt4c** | ASH | ASH | GLH | ASH | ASH | ASH | GLH | GLH | ASH | ASH | GLH | ASH | ASH | ASH | ASH |  |  |  |  |  |  |  |  |  |  |  |  |
| **1,61** | 65 | 76 | 85 | 87 | 137 | 147 | 149 | 161 | 164 | 170 | 172 | 177 | 178 | 190 | 193 |  |  |  |  |  |  |  |  |  |  |  |  |
| **wt6c** | ASH | GLH | ASH | ASH | ASH | GLH | GLH | ASH | ASH | GLH | ASH | ASH | ASH | ASH |  |  |  |  |  |  |  |  |  |  |  |  |  |
| **1,47** | 76 | 85 | 87 | 137 | 147 | 149 | 161 | 164 | 170 | 172 | 177 | 178 | 190 | 193 |  |  |  |  |  |  |  |  |  |  |  |  |  |
| **wt7c** | ASH | GLH | ASH | GLH | ASH | ASH | GLH | GLH | ASH | ASH | GLH | ASH | ASH | ASH | ASH |  |  |  |  |  |  |  |  |  |  |  |  |
| **1,46** | 76 | 85 | 87 | 136 | 137 | 147 | 149 | 161 | 164 | 170 | 172 | 177 | 178 | 190 | 193 |  |  |  |  |  |  |  |  |  |  |  |  |
| **wt8c** | ASH | GLH | ASH | ASH | GLH | ASH | GLH | GLH | ASH | ASH | GLH | ASH | ASH | ASH | ASH |  |  |  |  |  |  |  |  |  |  |  |  |
| **1,56** | 76 | 85 | 87 | 137 | 140 | 147 | 149 | 161 | 164 | 170 | 172 | 177 | 178 | 190 | 193 |  |  |  |  |  |  |  |  |  |  |  |  |
| **wt9c** | ASH | ASH | GLH | ASH | GLH | ASH | ASH | GLH | GLH | ASH | ASH | GLH | ASH | ASH | ASH | ASH |  |  |  |  |  |  |  |  |  |  |  |
| **1,62** | 61 | 65 | 73 | 76 | 85 | 137 | 147 | 149 | 161 | 164 | 170 | 172 | 177 | 178 | 190 | 193 |  |  |  |  |  |  |  |  |  |  |  |
| **wt4d** | ASH | ASH | GLH | ASH | ASH | ASH | GLH | GLH | GLH | ASH | ASH | GLH | ASH | ASH | ASH | ASH |  |  |  |  |  |  |  |  |  |  |  |
| **1,31** | 65 | 76 | 85 | 87 | 137 | 147 | 140 | 149 | 161 | 164 | 170 | 172 | 177 | 178 | 190 | 193 |  |  |  |  |  |  |  |  |  |  |  |
| **canonical (auto)** |  |  |  |  |  |  |  |  |  |  |  |  |  |  |  |  |  |  |  |  |  |  |  |  |  |  |  |
| **1,52** |  |  |  |  |  |  |  |  |  |  |  |  |  |  |  |  |  |  |  |  |  |  |  |  |  |  |  |
