# Supplementary material for: Molecular dynamics and structure function analysis show that substrate binding and specificity are major forces in the functional diversification of Eqolisins
Source: BMC Bioinformatics. 2018 Sep 24;19:338. doi: 10.1186/s12859-018-2348-2 (PMC6154417; doi:10.1186/s12859-018-2348-2)
Supplement: Supplementary file 3 — Table resuming TCS and SNAP analyses. Each of the removed sequences was tested by TCS. Consensus score was 79 and the lowest scoring accepted score was 51. All dubious mutations were analyzed by means of SNAP using 2IFW as a template. E indicates the prediction is that the mutation has an effect on enzyme activity, N means the mutation is predicted to be neutral. NA: Not applicable. (DOC 41 kb) [file 12859_2018_2348_MOESM3_ESM.doc]

|  | **Substitutions** | | | **TCS** | **SNAP** | | |
| --- | --- | --- | --- | --- | --- | --- | --- |
| **Seq name** | **n** | **WT aa** | **Var aa** | **Score** | **Effect** | **Score** | **Accuracy (%)** |
| **APf0000001** | 43 | D | S | 20 | E | 89 | 91 |
|  | 44 | G | N |  | E | 89 | 91 |
|  | 105 | L | Y |  | E | 60 | 80 |
| **BCa0000005** | 105 | L | Y | 51 | E | 74 | 85 |
| **EFAo000001** | 6 | W | L | 76 | E | 90 | 95 |
|  | 8 | G | S |  | E | 77 | 85 |
|  | 41 | G | E |  | E | 92 | 95 |
|  | 43 | D | S |  | E | 89 | 91 |
|  | 44 | G | S |  | E | 88 | 91 |
|  | 71 | Y | F |  | N | -4 | 53 |
| **EFAo000003** | 6 | W | GAP | 76 | NA |  |  |
|  | 8 | G | GAP |  | NA |  |  |
|  | 41 | G | P |  | E | 94 | 95 |
|  | 43 | D | K |  | E | 95 | 95 |
|  | 44 | G | A |  | E | 84 | 91 |
|  | 71 | Y | F |  | N | -4 | 53 |
| **EFBso00001** | 44 | G | S | 71 | E | 88 | 91 |
|  | 51 | I | Q |  | E | 76 | 85 |
|  | 71 | Y | F |  | N | -4 | 53 |
|  | 72 | P | S |  | E | 67 | 80 |
| **EFCm000002** | 71 | Y | W | 52 | N | -11 | 57 |
|  | 72 | P | Q |  | E | 54 | 75 |
| **EFCm000003** | 71 | Y | W | 47 | N | -11 | 57 |
|  | 72 | P | K |  | E | 66 | 80 |
| **EFMac00003** | 71 | Y | W | 49 | N | -11 | 57 |
|  | 72 | P | K |  | E | 66 | 80 |
| **EFMan00003** | 71 | Y | W | 51 | N | -11 | 57 |
|  | 72 | P | K |  | E | 66 | 80 |
| **EFPde00001** | 71 | Y | W | 51 | N | -11 | 57 |
|  | 72 | P | K |  | E | 66 | 80 |
| **EFPf000001** | 67 | W | A | 69 | E | 79 | 85 |
|  | 71 | Y | L |  | E | 69 | 80 |
| **EFSma00001** | 44 | G | R | 66 | E | 95 | 95 |
|  | 71 | Y | V |  | E | 59 | 75 |
| **EFTa000006** | 51 | I | P | 78 | E | 87 | 91 |
|  | 71 | Y | V |  | E | 59 | 75 |
| **EFTs000011** | 43 | D | G | 74 | E | 87 | 91 |
|  | 44 | G | GAP |  | NA |  |  |
| **EFTs000012** | 43 | D | G | 73 | E | 87 | 91 |
|  | 44 | G | GAP |  | NA |  |  |
