# Supplementary figures and images for: Molecular dynamics and structure function analysis show that substrate binding and specificity are major forces in the functional diversification of Eqolisins
Source: BMC Bioinformatics. 2018 Sep 24;19:338. doi: 10.1186/s12859-018-2348-2 (PMC6154417; doi:10.1186/s12859-018-2348-2)

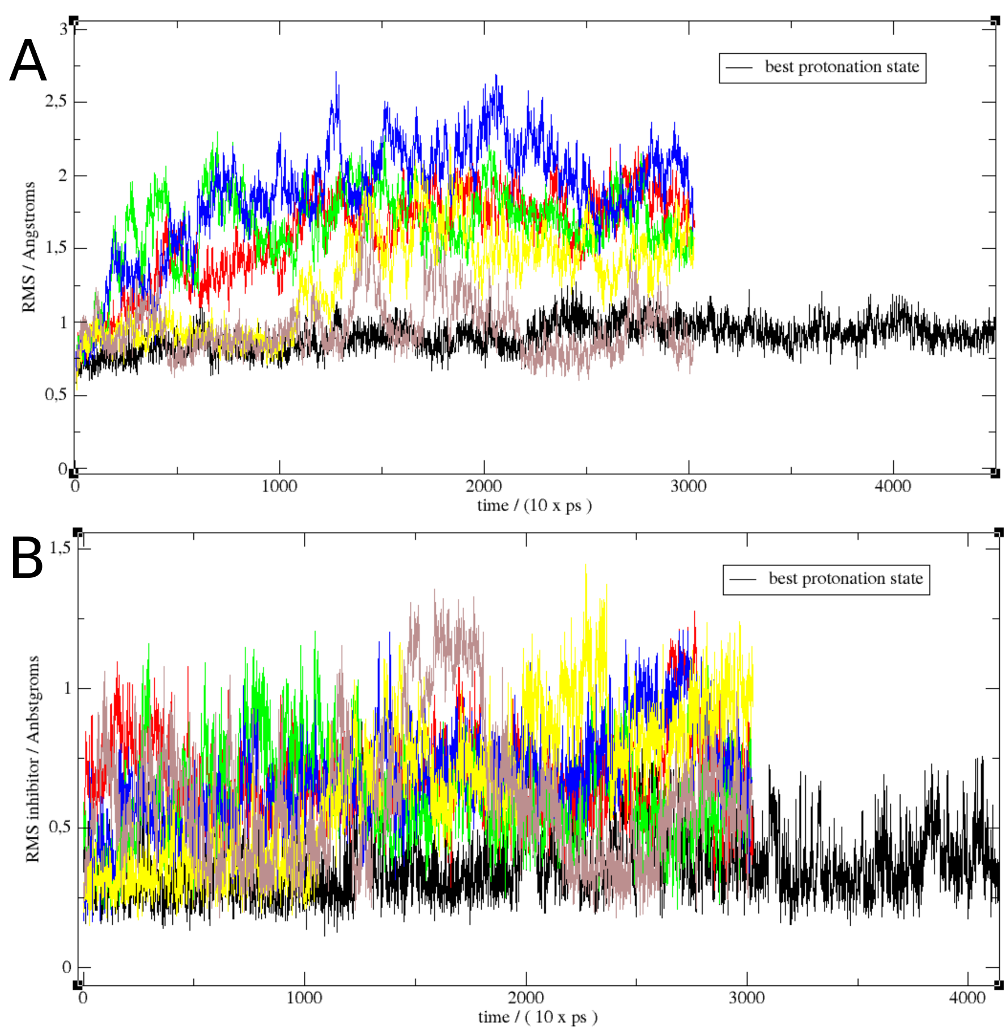

Supplement: Supplementary file 4 — Molecular Dynamics Simulation determining best protomer state. (A) Backbone RMSD of the best protomer for the WT compared to five of the other best. (B) Backbone RMSD of substrate analog inhibitor for the best protomer for the WT compared to five of the other best. (TIF 830 kb) [file 12859_2018_2348_MOESM4_ESM.tif]

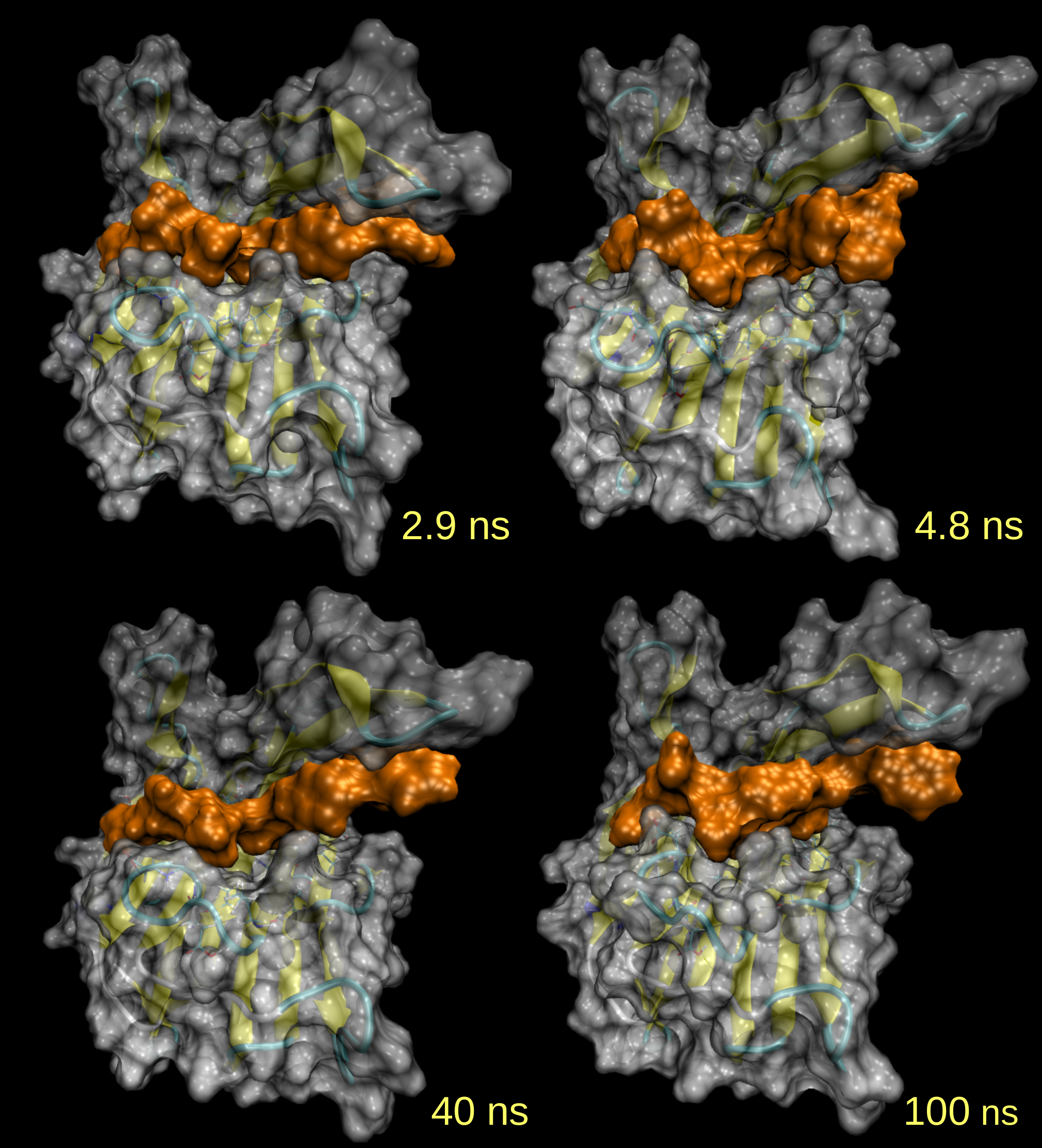

Supplement: Supplementary file 5 — (B) Cartoons of the GAx4 mutant at 2.9, 2.4, 40 and 100 ns of simulation showing displacement of the β-loop relative to the inhibitor (solid golden surface). (TIF 3478 kb) [file 12859_2018_2348_MOESM5_ESM.tif]

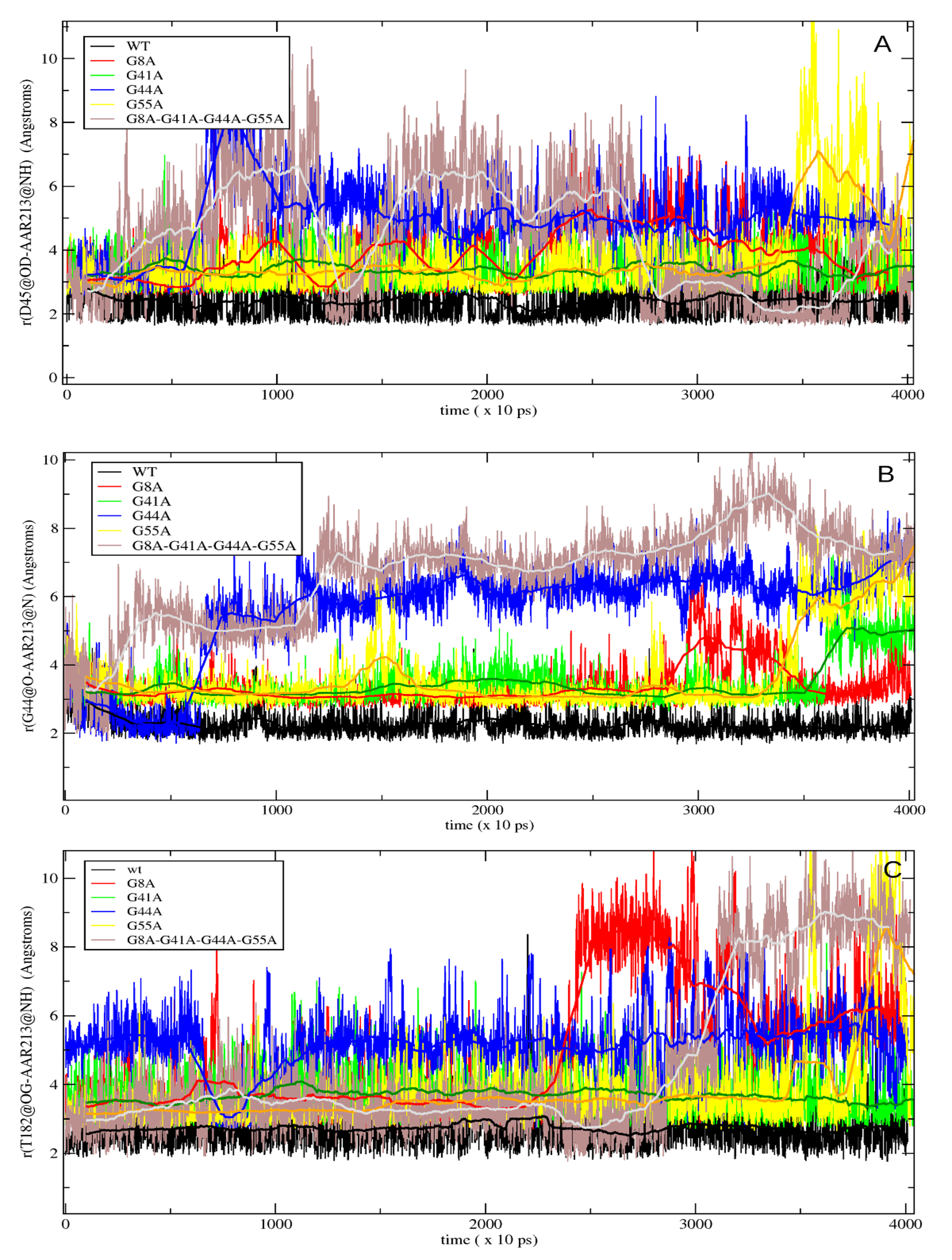

Supplement: Supplementary file 6 — Analyses of the changes in contacts for each individual GxA mutant, as compared to the WT. (A) The salt bridge D45-AAR213 is weakened for G41A and G55A and depleted for G8 and G55 (as in the case of GAx4). (B) The H-bond to AAR213 from the backbone G41 (or A41) oxygen is lost for G41A and weakened in the other three single mutants. (C) The H-bond involving the T182@OG is weaker than in the WT for the case of G41A and depleted or lost for G8, G44 and G55. Smooth thick lines are 200 ps running averages. (TIF 4486 kb) [file 12859_2018_2348_MOESM6_ESM.tif]

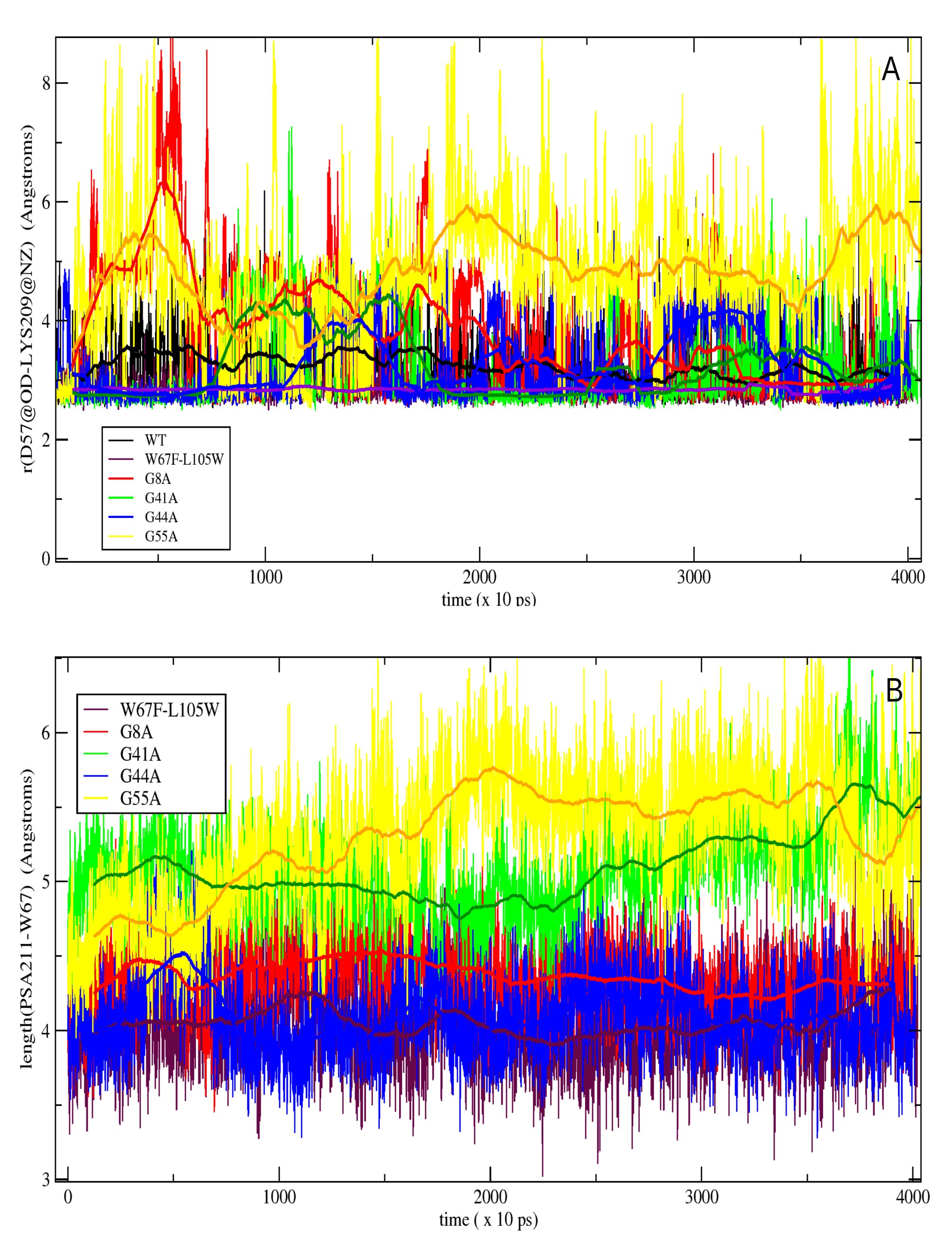

Supplement: Supplementary file 7 — Analyses of the changes in contacts for each individual GxA mutant compared to either WT or W67F-L105 double mutant. (A) The salt bridge between D57 and LYS209 of the inhibitor (which is tightest in the case of W67F-L105W) is depleted in the four GxA substitutions, especially for G8A and G55A. (B) Also the π-π stacking with PSA211 results weaker in the individual mutants G8A and G41A and practically absent in G55A. Smooth thick lines are 200 ps running averages. (TIF 3253 kb) [file 12859_2018_2348_MOESM7_ESM.tif]

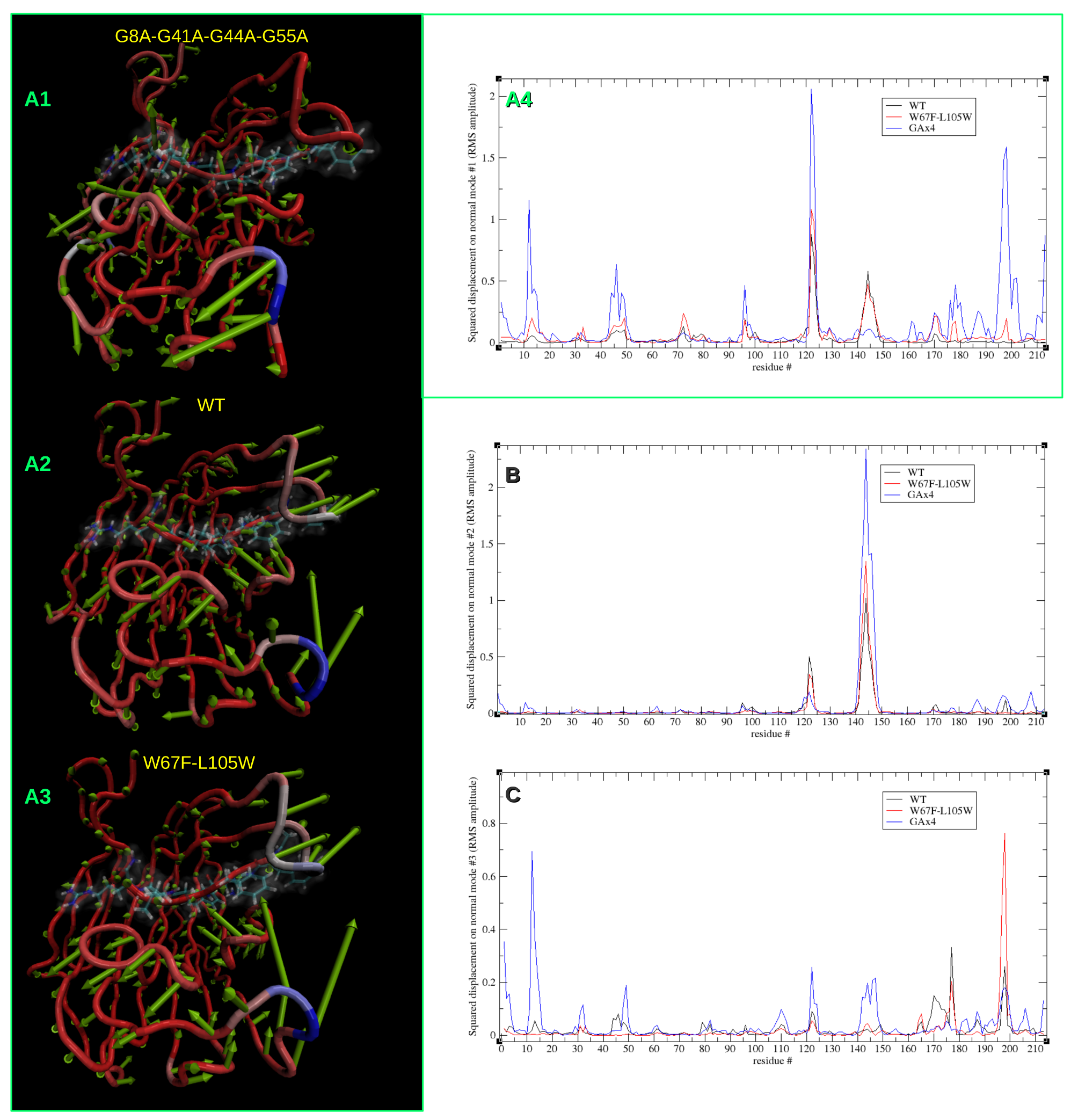

Supplement: Supplementary file 8 — (A1-A3) Ca vectors of the first essential normal mode from PCA analysis for the GAx4, WT and W67F-L105W species. (A4) the squared displacement of each residue in the first mode. B-C squared displacement of each residue on the next two modes. (TIF 1669 kb) [file 12859_2018_2348_MOESM8_ESM.tif]

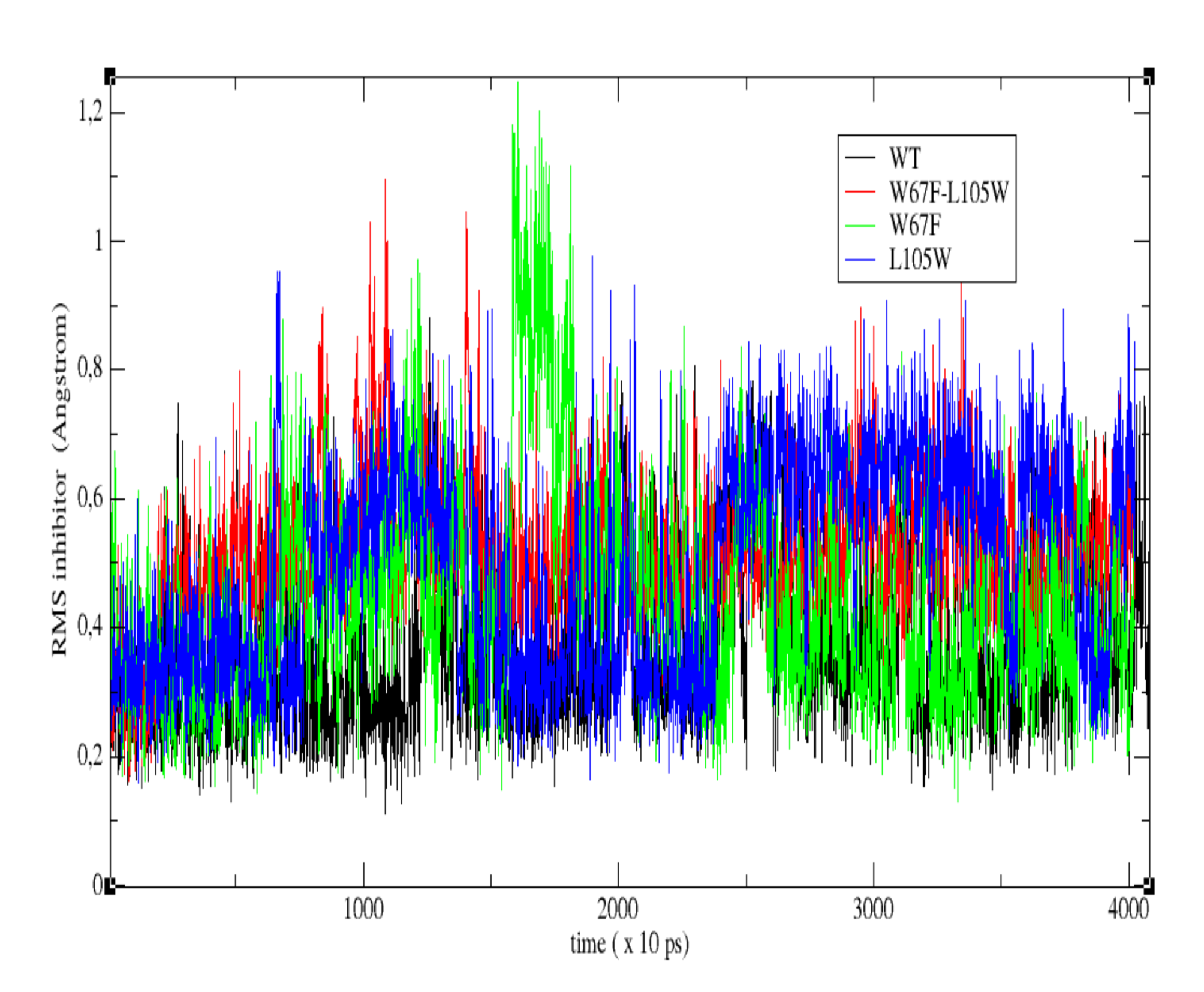

Supplement: Supplementary file 9 — RMSD of the inhibitor residues for the WT, W67F, L105W and the double mutant. (TIF 2432 kb) [file 12859_2018_2348_MOESM9_ESM.tif]

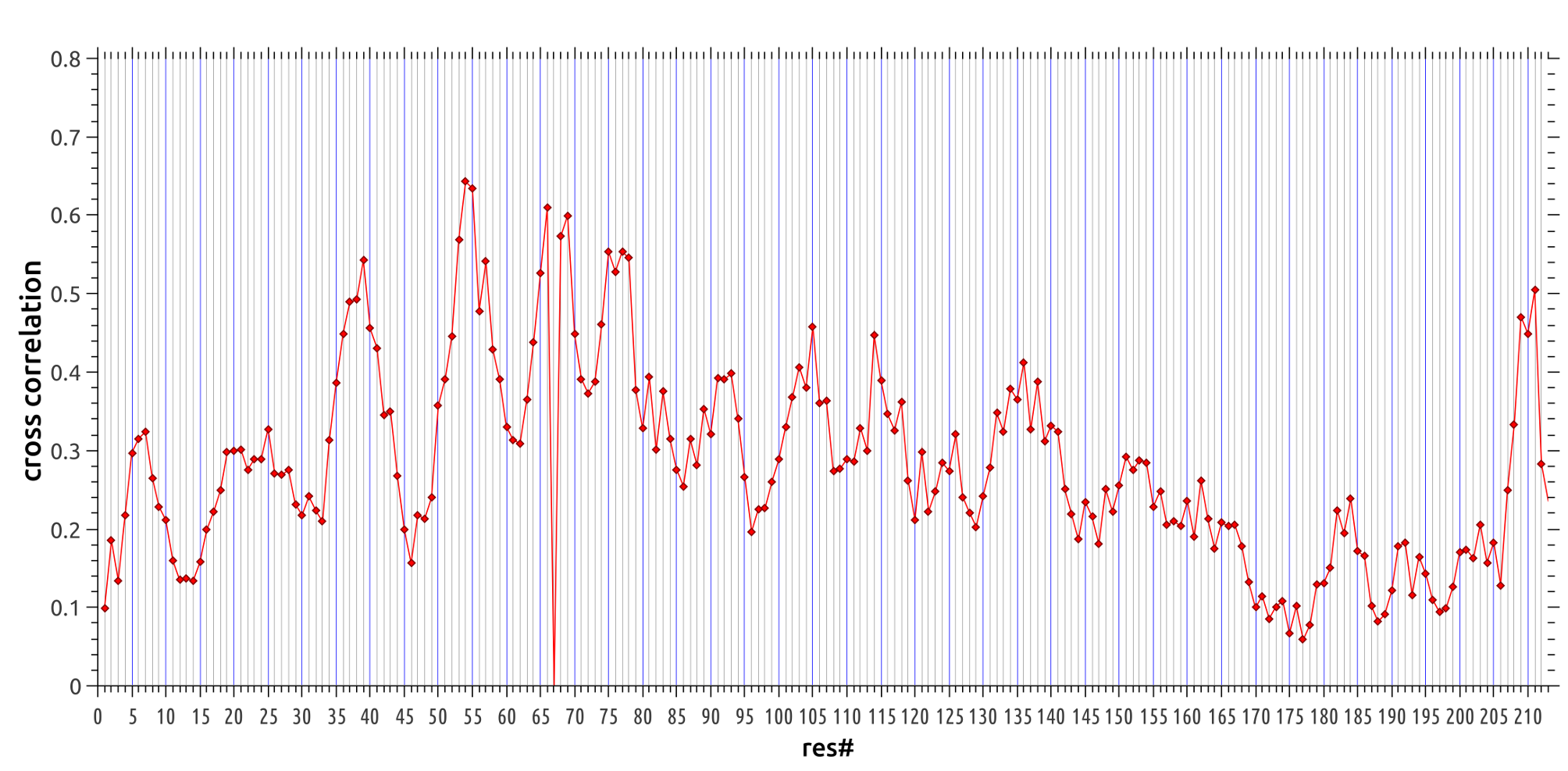

Supplement: Supplementary file 10 — Squared cross correlation function of W67 against all other residues (wt trajectory). (TIF 1550 kb) [file 12859_2018_2348_MOESM10_ESM.tif]

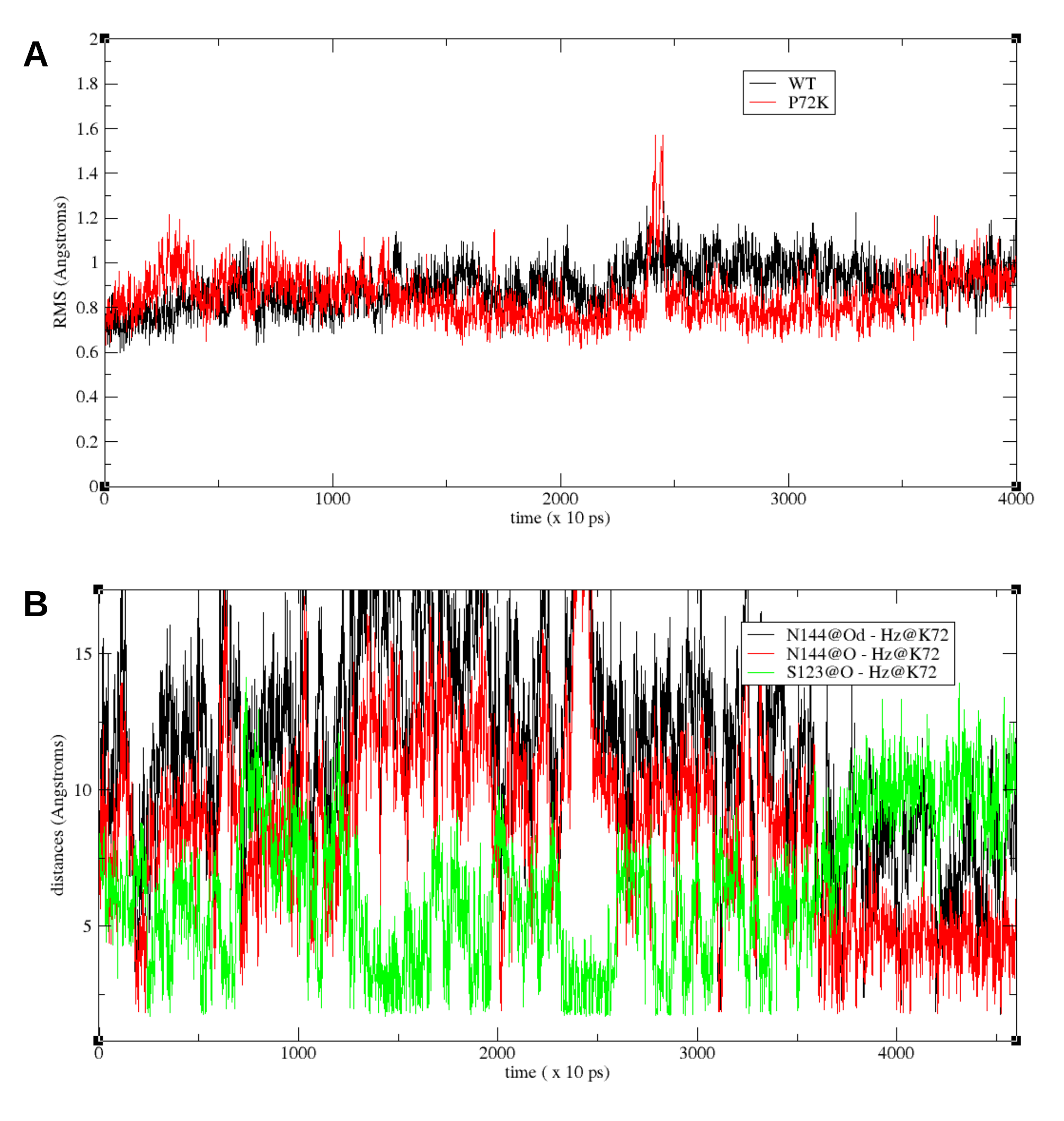

Supplement: Supplementary file 11 — Backbone RMSD comparison for the WT and the P72K mutant. (TIF 2162 kb) [file 12859_2018_2348_MOESM11_ESM.tif]

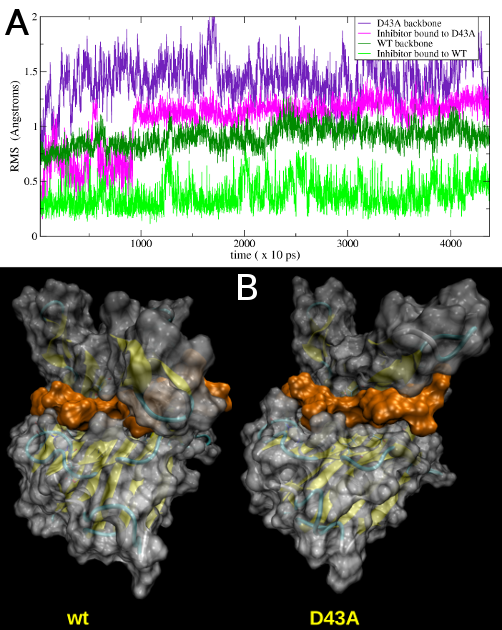

Supplement: Supplementary file 12 — Molecular Dynamics Analyses of D43A mutant. (A) Comparison of the backbone RMSD of the WT and the D43A mutant. (B) Cartoons of WT and D43A mutant showing displacement of the ß-loop relative to the inhibitor (solid golden surface). (TIF 397 kb) [file 12859_2018_2348_MOESM12_ESM.tif]

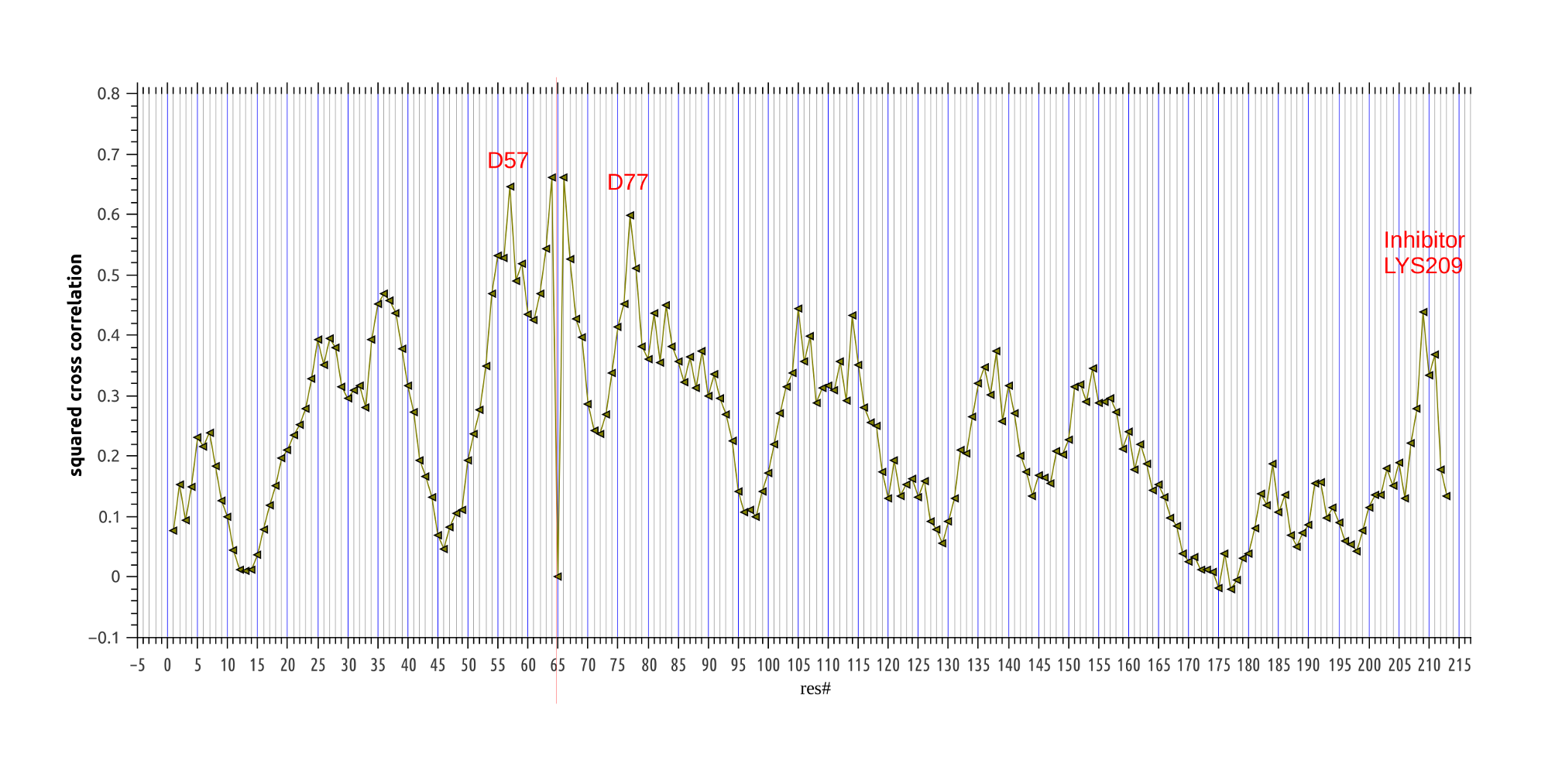

Supplement: Supplementary file 16 — Squared cross correlation function of D65 against all other residues (WT trajectory), showing main peaks at D57 and D77 as well as inhibitors LYS209, which are involved in a network of H-bond and salt bridges network. (TIF 1361 kb) [file 12859_2018_2348_MOESM16_ESM.tif]
